# Stoichiometric Selective Carbonylation of Methane to Acetic Acid by Chemical Looping

**DOI:** 10.1021/acscatal.4c07095

**Published:** 2025-02-05

**Authors:** Yinghao Wang, Chunyang Dong, Mariya Shamzhy, Maya Marinova, Zhengxiao Guo, Yury G. Kolyagin, Jeremie Zaffran, Andrei Khodakov, Vitaly V. Ordomsky

**Affiliations:** † UCCS−Unité de Catalyse et Chimie du Solide, Université de Lille, CNRS, Centrale Lille, ENSCL, Université d’Artois, UMR, 8181 Lille, France; ‡ Department of Chemistry, The University of Hong Kong, 085295576619 Hong Kong, China; § Department of Physical and Macromolecular Chemistry, Faculty of Science, Charles University, Hlavova 2030/8, 12843 Prague, Czech Republic; ∥ Institut Michel-Eugène Chevreul, 59655 Villeneuve-d’Ascq, France; ⊥ Eco-Efficient Products and Processes Laboratory (E2P2L), IRL 3464 CNRS-Syensqo, 3966 Jin Du Road, Xin Zhuang Ind. Zone, 201108 Shanghai, China

**Keywords:** methane activation, chemical loop, oxidative carbonylation, acetic acid, high selectivity

## Abstract

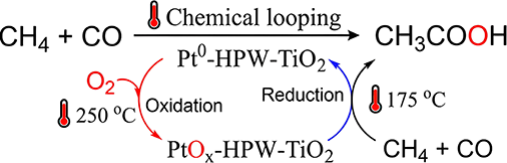

The conversion of methane to valuable products is one of the main challenges of modern chemistry. Acetic acid (AcOH) is a key chemical reagent in industry, produced nowadays by the carbonylation of methanol over homogeneous Rh and Ir catalysts. Here, we propose a stepwise chemical looping approach for the highly selective stoichiometric synthesis of AcOH by carbonylation of methane with CO using single-site Pt over isolated phosphotungstic anions on a titania support (Pt-HPW-TiO_2_). The reaction proceeds by methane activation, which coincides with the reduction of initially oxidized Pt species in the presence of CO at 423 K and results in surface acetates attached to TiO_2_. Subsequent hydrolysis by water at ambient temperature results in the synthesis of AcOH in a stoichiometric amount corresponding to 1.5 Pt. Spent Pt-HPW-TiO_2_ is restored to the initial state by subsequent calcination in air. This approach provides an opportunity for the selective synthesis of AcOH (>99% in liquid phase) from methane, carbon monoxide, and air. A high concentration of AcOH (1.1 wt %) in an aqueous solution can be obtained at a high conversion of methane (4.5%).

## Introduction

Methane is the main component of natural gas, with worldwide production of about 4 trillion cubic meters. Methane is a more potent greenhouse gas than CO_2_. Currently, an extensive amount of waste methane generated from remote oil fields is directly flared, resulting in the emission of an equivalent amount of CO_2_ into the atmosphere.^1,2^ Instead, via decentralized small-scale systems, methane valorization to produce value-added chemicals under mild conditions can be an acceptable solution to use these waste carbon resources and reduce carbon emission.^3^ Nonetheless, the extreme inertness of the methane molecule makes its functionalization challenging, calling for innovative approaches to activate methane under mild conditions and to selectively produce value-added concentrated single products that fit industrial demands.^4−8^

As an important chemical feedstock, acetic acid (AcOH) has a global annual demand of around 18 million tons and is used as a reagent in the manufacturing of fine chemicals such as polyvinyl acetate. In industry, AcOH is produced by the sequential fermentation of sugars and oxidation of ethanol, Pt- and Pd-catalyzed oxidation of ethylene and acetaldehyde, and homogeneous Rh- or Ir-catalyzed methanol carbonylation,^13^ with the last accounting for the highest market share globally.^14^ However, despite the high yield of methanol carbonylation, the use of methanol and iodide additives has made this process less attractive, in terms of cost and environmental impact. The use of methane as a raw material for the direct one-step synthesis of AcOH holds greater significance in both economic and sustainable aspects.^9,15−18^

**Figure 1:**
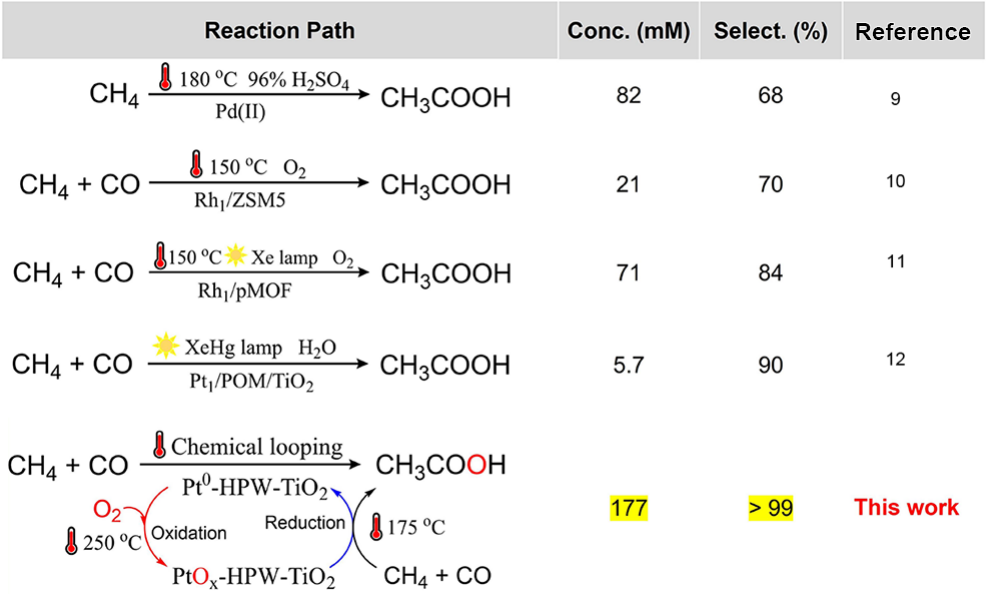
Different routes to produce acetic acid via direct methane oxidation or oxidative carbonylation.^9−12^

The pioneering study of Periana et al. reports an oxidative condensation path to transform methane into AcOH within an H_2_SO_4_ medium.^9^ Despite the high AcOH concentration achieved, the harsh reaction conditions make the process challenging to operate (Figure 1, 1st row). Catalysts with isolated Rh single-atom sites and porous support demonstrate high efficiency in AcOH production using O_2_ as an oxidant (Figure 1, 2nd and 3rd row).^10,11,19^ However, the inevitable overoxidation of AcOH by O_2_ severely restricts the maximum achievable AcOH yield and concentration. Recently, we reported a direct photocatalytic synthesis of AcOH from methane and CO at ambient temperature over a nanocomposite of TiO_2_ and ammonium phosphotungstic polyoxometalate subnano clusters containing Pt single-atom species, using water as an oxidizing agent.^12^ A detailed mechanistic investigation shows that the synthesis of acetic acid proceeds via photocatalytic oxidative carbonylation of methane over the Pt single-atom sites, with methane activation facilitated by water-derived hydroxyl radicals. Though the selectivity of AcOH reaches 90% in long-term reactions (Figure 1, 4th row), the relatively slow reaction kinetics, low AcOH concentration, and operational risks due to the use of UV irradiation prompt us to develop a thermochemical reaction operative under mild conditions.

Chemical looping is a cyclic chemical conversion approach, where a chemical reagent (substrate) is periodically exposed and reacts with a solid material, yielding the target reaction product. During the reaction, the solid material undergoes multiple chemical transformations. The target product is then removed from the reactor, and the solid material is regenerated for another reaction cycle. Chemical looping can be also used for conducting reactions, which are not thermodynamically favorable under steady-state conditions or kinetically limited such as methane conversion to valuable products.^4,20,21^ Since the thermodynamics is quite favorable for AcOH synthesis via direct methane carbonylation in the presence of oxygen (eq 1), the key challenge of this reaction lies in slowing the overoxidation of acetic acid and the water gas shift (WGS) reaction, resulting in excessive formation of CO_2_.
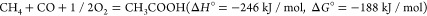
1

Hereby, we propose to use the chemical looping strategy to overcome the overoxidation issue of AcOH by conducting methane carbonylation without gaseous oxygen. We utilize an initially oxidized single-site Pt over isolated phosphotungstic anions with TiO_2_ support (Pt-HPW-TiO_2_) to convert methane and CO selectively to surface acetate. Methane activation has been reached at a relatively low temperature (175 °C) and coincides with the reduction of PtO_*x*_ species. Subsequent hydrolysis by the addition of water results in the selective synthesis of AcOH. Significantly, via this chemical looping approach, we managed an unprecedented high AcOH concentration of 177 mM with over 99% AcOH selectivity in the liquid phase (Figure 1, 5th row).

## Methods

### Chemicals

Aluminum oxide (Al_2_O_3_) was purchased from Alfa Aesar. Other chemicals including tetraammineplatinum(II) nitrate ([Pt(NH_3_)_4_](NO_3_)_2_, ≥99.995% trace metals basis), tetraamminepalladium(II) nitrate ([Pd(NH_3_)_4_](NO_3_)_2_, 10 wt % in H_2_O), rhodium(III) nitrate hydrate (Rh(NO_3_)_3_·*x*H_2_O, ∼36% rhodium basis), cobalt(II) nitrate hexahydrate (Co(NO_3_)_2_·6H_2_O, ≥99.999% trace metals basis), copper(II) nitrate hydrate (Cu(NO_3_)_2_·*x*H_2_O, ≥99.999% trace metals basis), niobium(V) oxide (Nb_2_O_5_, ≥99.99% trace metals basis), zirconium(IV) oxide (ZrO_2_, powder, 5 μm, ≥99% trace metals basis), silicon dioxide (SiO_2_, nano powder, 5–20 nm particle size, ≥99.5% trace metals basis), cerium(IV) oxide (CeO_2_, powder, <5 μm, 99.9% trace metals basis), phosphotungstic acid hydrate (H_3_[P(W_3_O_10_)_4_]·*x*H_2_O, abbreviated as HPW, reagent grade), phosphomolybdic acid hydrate (H_3_[P(Mo_3_O_10_)_4_]·*x*H_2_O, abbreviated as HPM, ACS reagent), tungstosilicic acid hydrate (H_4_[Si(W_3_O_10_)_4_]·*x*H_2_O, abbreviated as HSiW, ≥99.9% trace metals basis), titanium dioxide (TiO_2_, P25, ≥99.5% trace metals basis), dimethyl sulfoxide ((CH_3_)_2_SO, DMSO, anhydrous, ≥99.9%), and acetic acid (CH_3_COOH, AcOH, ACS reagent, ≥99.8%) were all purchased from Sigma-Aldrich. All chemicals were used as received without further purification.

### Synthesis of Nanocomposite Materials

The metal-heteropolyacid (HPA)-TiO_2_ nanocomposites were prepared by the two-step impregnation of TiO_2_. During the first impregnation, a fixed amount of TiO_2_ was suspended in a water solution of heteropolyacid (HPA) hydrate. The heteropolyacid-to-TiO_2_ ratio varied from 0.15 to 1.2. After water evaporation, the resulting sample was washed 2 times and then dried at 80 °C for 12 h. The obtained HPA-TiO_2_ composites were subsequently impregnated with aqueous solutions of the relevant metal salts. The target metal content in the final sample was 0.2 wt %. Finally, after a 2 h calcination at 250 °C in static air, the ternary-phase composite of metal-HPA-TiO_2_ was obtained. The composites with different metals (i.e., M-HPA-TiO_2_, M = Pd, Rh, Co, and Cu) or supports (i.e., Al_2_O_3_, Nb_2_O_5_, ZrO_2_, SiO_2_, and CeO_2_) as well as metal-free HPA-support composites were prepared similarly, by simply replacing either the metal precursor or support. Moreover, the reference Pt-TiO_2_ composite was prepared via the strong electrostatic adsorption (SEA) method, with a theoretical Pt loading of 0.2 wt %. Typically, 500 mg of TiO_2_ was dispersed in water and subjected to sonication treatment. Then, [Pt(NH_3_)_4_](NO_3_)_2_ powder was added to the TiO_2_-water suspension under vigorous stirring at room temperature. After 2 h of stirring, the sample precursor was washed with DI water and dried at 80 °C overnight. Lastly, the dried sample was calcined at 250 °C in static air for 2 h.

### Characterization Techniques

Scanning transmission electron microscopy (STEM) was performed on a TITAN Themis 300 S/TEM microscope equipped with a probe aberration corrector and monochromator, allowing a spatial resolution of 70 pm and an energy resolution of 150 meV, a super-X windowless 4 quadrant SDD (silicon drift detector) detection system for STEM-EDX mapping, and several annual dark field detectors. The measurements were performed with a spot size of about 500 pm, a semiconvergence angle between 20 mrad, and a probe current of approximately 100 pA. For the HAADF images, collection angles were chosen between 50 and 200 mrad. In situ FTIR spectra were recorded on a Nicolet 6700 FTIR Spectrometer (Thermo Fisher Scientific) with a mercury cadmium telluride detector. Before analysis, a 40 mg of the sample was compressed into a wafer with a diameter of 13 mm. Then, the sample wafer was transferred into the in situ reaction cell and degassed under high vacuum (<10^–5^ Torr) at room temperature for 60 min. Afterward, a mixed CO and CH_4_ gas (1 bar, 1/15, v/v) was introduced into the reaction cell, and such condition was maintained in the dark for 30 min. The catalyst was heated to the required temperature. It should be noted that once the sample wafer was loaded into the reaction cell, the FTIR spectrum was recorded continuously (32 scans at a resolution of 4 cm^–1^). The FTIR analysis was conducted by introducing the respective probe molecules, CO (20 mbar) or pyridine (7 mbar), into the in situ cell.

X-ray photoelectron spectroscopy (XPS) was performed on a Kratos Axis Ultra DLD photoelectron spectrometer using monochromatic Al Ka (1486.7 eV) X-ray irradiation. High-resolution spectra were collected using an analysis area of 300 mm and 700 mm and a 20 eV pass energy. The binding energy of the photoemission spectra was calibrated to the Ti 2p_3/2_ peak with a binding energy of 458.6 eV. Model experiments on the interaction of TiO_2_ and HPW with AcOH were performed using 50 mg of TiO_2_ and HPW treated with 50 μL of AcOH.

### Chemical Looping

The reaction was performed in a 50 mL autoclave reactor. In a typical batch, 50 mg of sample powder was first put into the reactor. The reactor was evacuated by a vacuum pump and charged sequentially with CO and CH_4_ to a designated pressure (1–16 bar, absolute pressure).^22^ For comparison purposes, the catalyst was exposed to a mixed CO (1 bar) + Ar (15 bar) atmosphere. Throughout the reaction, the reactor was kept at 175 °C on a hot plate stirrer without stirring. After the reaction, the gaseous products were analyzed by a gas chromatograph (Agilent 8860) equipped with PoraBOND Q and ShinCarbon ST 100/120 columns as well as a thermal conductivity detector and a flame ionization detector. To extract the produced acetic acid, 1 mL of deionized (DI) water was added to the reactor for hydrolysis of the product-sample adduct at 40 °C for 30 min with stirring. Then, the extracted liquid product was analyzed by ^1^H nuclear magnetic resonance (NMR) spectroscopy. One mL solution was separated by a centrifuge and mixed with 0.1 mL of DMSO/D_2_O solution (1/2000, v/v, DMSO is the internal standard).

For the regeneration experiments and cyclic test, after hydrolysis treatment, the isolated sample was dried in an oven at 80 °C overnight. Lastly, the dried sample was further calcined at 250 °C in static atmospheric air in the autoclave reactor for 2 h for the next cycle. For the synthesis of highly concentrated acetic acid, we used a cold trap to condense and collect the water vapor evaporated from the heated catalyst, which was wetted with 0.1 g of water. For the high-conversion methane experiment, we used 1 g of the sample in a gas mixture (1 bar CH_4_, 1/15 bar CO, and 15 bar N_2_ at room temperature) at 175 °C for 2 hours in a sealed 2 mL metal reaction tube.

The liquid-phase selectivities of AcOH were calculated based on the mole numbers of all liquid products (eq 2). The concentrations of AcOH were calculated based on the mass of AcOH and solvent (eq 3). The conversion of methane was calculated based on the mole numbers of AcOH and methane in the reactor considering that acetic acid contains one methane molecule (eq 4).
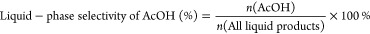
2
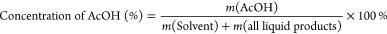
3
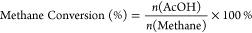
4

### NMR Measurements

Solid-state NMR measurements were performed on a Bruker AVANCE-NEO 9.4 T NMR equipped with 3.2 and 4.0 mm HXY DVT Bruker MAS probes, with working frequencies of ^1^H, ^13^C, and ^17^O equal to 400.11, 100.6, and 54.24 MHz, respectively. The samples were carefully packed into corresponding ZrO_2_ MAS rotors under a dry argon atmosphere directly after the described procedures, without any contact with oxygen or atmospheric moisture.

^1^H–^13^C CP-MAS spectra were recorded with a rotation speed of 12 kHz, a relaxation delay of 1.5 s, a contact time of 2.0 ms, with ramp-shaped from 100 to 70% of power on the ^1^H-channel, and the number of scans from 10 to 40 thousand, depending on the sample. For ^1^H–^13^C CP-HETCOR NMR, a 1.0 ms contact time and a 1.0 s relaxation delay were used. 2D spectra were recorded with 55 *t*_1_—increments by 83.3 μs in States-TPPI mode and 768 scans per each step.

For ^1^H Hahn-echo (HE) MAS and ^1^H{^17^O} S-RESPDOR spectra, a 3.2 mm probe with a 20 kHz rotation speed was used. For ^1^H-detected sequences, 256 scans for each 1D spectrum were used, with a 3 s relaxation delay and preliminary presaturation. A 100 μs echo delay (2 rotor’s rotation periods) was applied for ^1^H HE/MAS spectra. In ^1^H{^17^O} S-RESPDOR experiments, *SR*4_1_^2^ recoupling blocks during the echo delays on the ^1^H-channel and a 75 μs (1.5 rotor’s rotation periods) rectangular saturation pulse with maximal available power on the ^17^O-channel were applied.^23^

### DFT Modeling

The theoretical work was carried out using VASP software,^24^ including Henkelman’s tools (nudged elastic band—NEB^25^ and the dimer^26^ methods) for the transition state (TS) search. Density functional theory (DFT) calculations were led in the GGA framework with the Perdew–Burke–Ernzerhof (PBE) exchange–correlation (XC) functional.^27^ The ion–electron interaction was addressed in the projected augmented wave (PAW) formalism,^28^ with a cutoff of 500 eV. While the electronic wave functions were converged with an energetic criterion of 10^–5^ eV, the structure geometries were relaxed with a force threshold of 0.05 eV/Å. All of the calculations were performed with a single-point *k*-mesh at the gamma center. The zero-point energy (ZPE) and entropy corrections were not considered. The Bader charge analysis was performed using the Henkelman's tools.^29^

Starting from a relaxed TiO_2_ bulk structure (anatase phase), we built a two-layer thickness slab according to the (101) plane reported to be among the most stable facets of this sample.^30^ While the surface is partially oxidized, our slab model is symmetric in the *z*-direction, hence avoiding any dipolar momentum to appear. Before depositing HPW with an embedded Pt single-atom sample at the TiO_2_ surface, the primitive cell was first replicated to give a (2 × 4) slab, to prevent interactions between the periodic images of the macrostructures. The polyoxometalate was located so that the Pt atom facets the surface and could operate in synergy with TiO_2_ during the process. In every situation, including the bare slab and the reactant/product states, we tested several configurations at the surface, and only the most stable ones were considered for the reactivity study. The initial TiO_2_ bulk and the Keggin anion structures were both taken from the online “American Mineralogist Crystal Structure Database”.

## Results and Discussion

### Synthesis of AcOH

First, methane and carbon monoxide were exposed to Pt-HPW-TiO_2_ in an aqueous phase in a batch reactor at different temperatures. A noticeable WGS was observed at temperatures higher than 100 °C, leading to the production of CO_2_ and hydrogen (Figure S1, SI). No product was detected in the aqueous phase. Then, methane and carbon monoxide were exposed to Pt-HPW-TiO_2_ in a dry state. The first cycle involved the treatment of 50 mg of the dry sample in the batch reactor with a mixture of CH_4_ and CO (CH_4_/CO = 15 at a total pressure of 16 bar) at 175 °C for 2 h. After the reaction, the gas phase contained much smaller amounts of CO_2_ (25 μmol/g_solid_), compared to the reaction involving the aqueous liquid phase (1801 μmol/g_solid_), due to catalyst reduction by CO (Figure S1, SI). This was confirmed by a test with only CO in the gas phase without methane (Figure S1, SI), which produced an even higher amount of CO_2_ than in the presence of methane, likely due to the consumption of catalyst active oxygen in reactions with methane. After analysis of the gas-phase products, the reactor was open, and 1 g of water was added to dissolve the product adsorbed over Pt-HPW-TiO_2_. The obtained aqueous solution, after filtering from the solid nanocomposite, was analyzed by NMR. The liquid phase contained only AcOH, with an amount of 15 μmol/g_solid_, which corresponds to about 1.5 of the molar amount of Pt atoms in the Pt-HPW-TiO_2_ samples. The solid sample was calcined in air at 250 °C before the next reaction cycle. The presence of air is important during calcination; calcination of the spent Pt-HPW-TiO_2_ in inert Ar does not result in the regeneration of the nanocomposite for methane carbonylation (Figure S2, SI).

**Figure 2:**
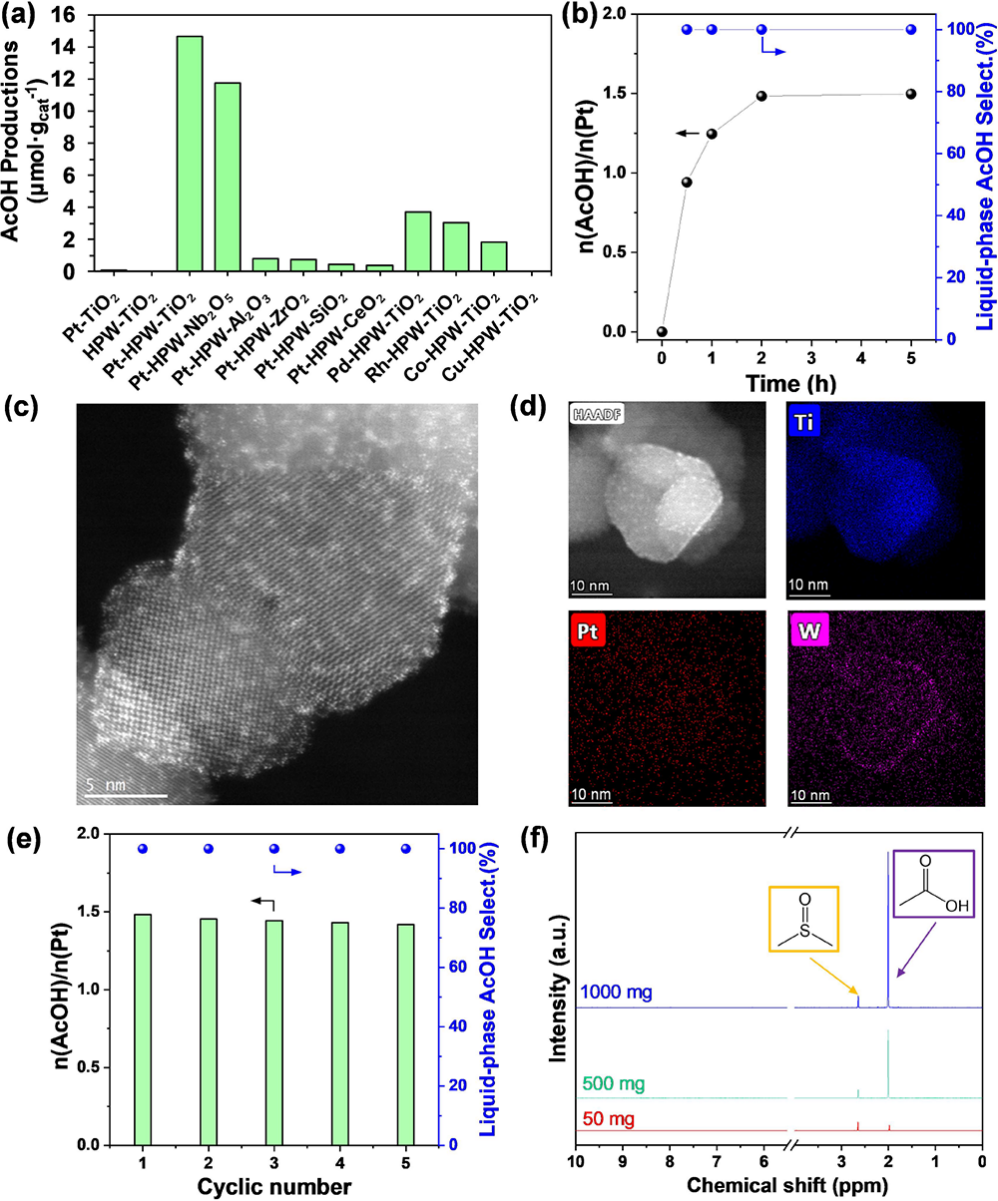
(a) Productivities of AcOH after hydrolysis over 50 mg of different samples treated by CH_4_ (15 bar) and CO (1 bar) at 175 °C for 2 h; (b) ratio AcOH to Pt and liquid-phase AcOH selectivities depending on the reaction time; (c) HAADF-STEM and (d) corresponding EDS-mapping images of the fresh Pt-HPW-TiO_2_; and (e) ratio AcOH to Pt over Pt-HPW-TiO_2_ in five regenerations by air and reaction cycles. (f)^1^H NMR analysis of AcOH produced over 50, 500, and 1000 mg of Pt-HPW-TiO_2_. Pt content of all the above samples is 0.2 wt %, and the weight ratio of HPW/TiO_2_ is 0.6.

Similar chemical looping tests were conducted with other solid samples (Figure 2a,b). It should be noted that only traces of AcOH have been detected over reference Pt-TiO_2_ and HPW-TiO_2_. The samples prepared using other metals such as Co, Cu, Ru, Rh, and Pd showed much lower AcOH productivity. Other supports like ZrO_2_, SiO_2_, CeO_2_, and Al_2_O_3_ also provided significantly lower amounts of AcOH in comparison with TiO_2_. Only niobium oxide (Nb_2_O_5_) demonstrates performance similar to that of TiO_2_ (Figure 2a).

The chemical looping strategy and recyclability of the solid nanocomposites were further verified by conducting several reaction cycles with intermediate regeneration by air calcination. The STEM images of the most active Pt-HPW-TiO_2_ before the reaction, after the reaction, and after regeneration (Figures 2c,d and S3–S7, SI) show some Pt agglomeration after the reaction and redispersion back into the atomically dispersed Pt state after regeneration. No leaching of heteropolyacid was observed after regeneration. Elemental analysis of the liquid phase used for extraction of acetic acid did not show the presence of HPW species. Indeed, intermediate calcination seems essential to stabilize HPW fragments over TiO_2_. Furthermore, Pt-HPW-TiO_2_ shows stable performance for five regenerations by air and reaction cycles, without a decrease in AcOH production and selectivity (Figure 2e). Furthermore, the looping concept allows the production of a concentrated product by increasing the amount of the solid nanocomposite sample and decreasing the amount of the aqueous phase used for the extraction of the reaction product. The synthesis of AcOH using 1 g of sample and 0.1 g of water for AcOH extraction results in the selective synthesis of a 1.1 wt % solution (Figure 2f), which is significantly higher than previously reported concentrations in the literature (Table S1, SI). We could achieve 4.5% methane conversion by using 1 g of sample in a gas mixture containing 1 bar CH_4_, 1/15 bar CO, and 15 bar N_2_ and operating at 175 °C for 2 h in a 2 mL metal reaction tube, whose both ends are sealed by two-way valves.

Variation in the type of commercial heteropolyacid in Pt-HPA-TiO_2_ composites reveals that phosphotungstic (HPW) acid shows the best productivity compared with phosphomolybdic (HPM) and silicotungstic (HSiW) counterparts (Figure S8a, SI). The increase in the content of HPW leads to a slight increase in the production of AcOH, reaching a maximum at a weight ratio at HPW/TiO_2_ of 0.6 (Figure S8b, SI). The Pt content has a crucial effect on AcOH production, with a linear increase in the range from 0.05 to 0.2 wt %, with approximately the same ratio of produced AcOH to Pt equal to 1.5. It is interesting to note that at a Pt content higher than 0.2 wt %, AcOH production decreases most probably due to the formation of Pt nanoparticles (Figure S8c, SI). Variation in temperature (Figure S8d, SI) shows that the reaction starts at 100 °C, with an increase in AcOH production until 175 °C and then decreases at higher temperatures. This decrease in AcOH productivity at higher temperatures can be attributed to the competitive oxidation of CO to CO_2_ instead of methane oxidative carbonylation to acetic acid.

**Figure 3:**
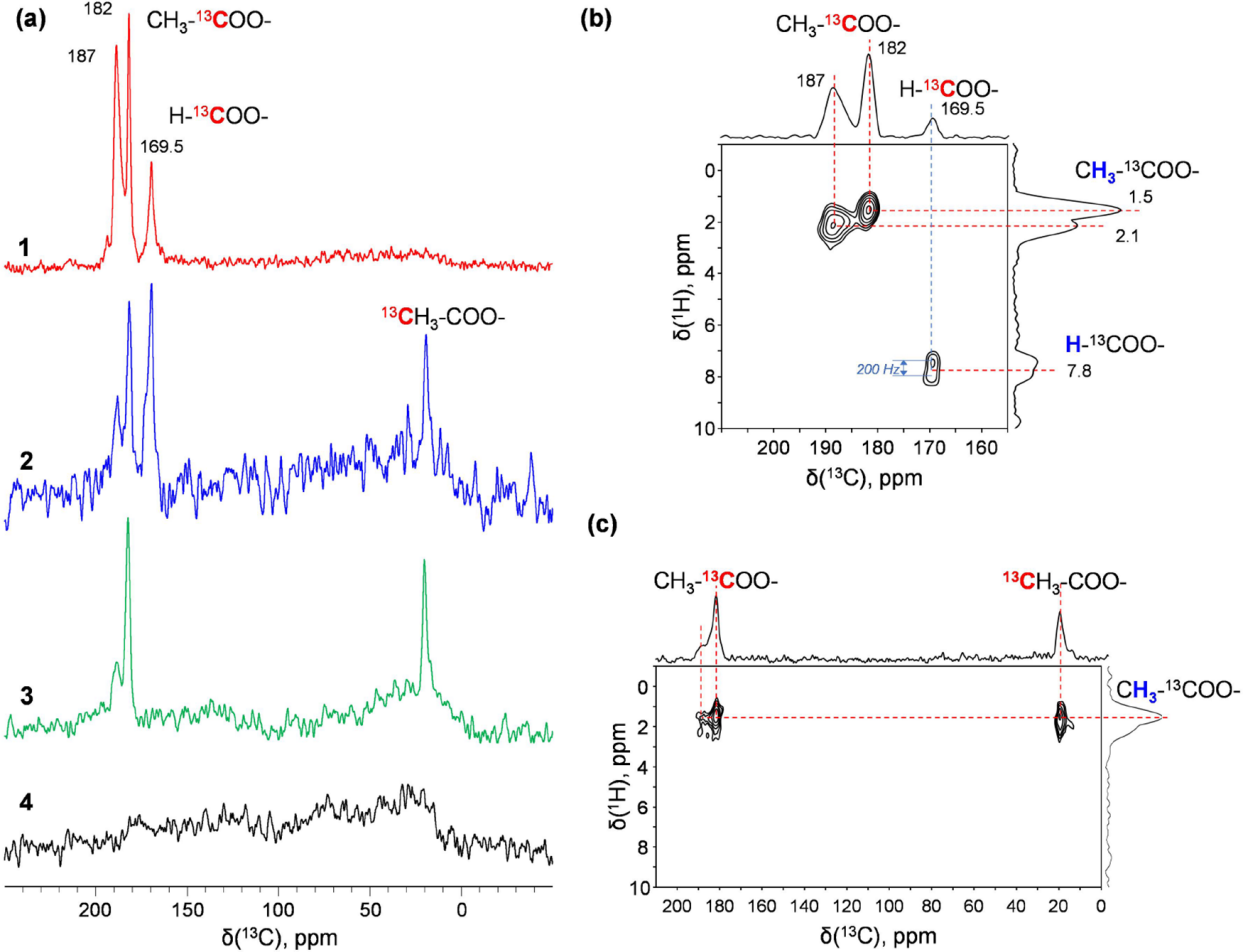
(a) ^1^H–^13^C CP/MAS NMR of Pt-HPW-TiO_2_ after treatment at 175 °C by (1) ^13^CO and CH_4_; (2) ^13^CO and ^13^CH_4_ with the following maintenance under an Ar atmosphere for 5 h and (3) 3 days at r.t. (4) Pt-HPW-TiO_2_ after treatment by ^13^CO and CH_4_, and subsequent hydrolysis by water; (b) 2D^1^H–^13^C CP-HETCOR NMR of Pt-HPW-TiO_2_ after treatment at 175 °C by ^13^CO and CH_4_; (c) 2D^1^H–^13^C CP-HETCOR NMR of Pt-HPW-TiO_2_ after treatment by ^13^CO and ^13^CH_4_ with the following maintenance under an Ar atmosphere for 3 days at r.t.

To confirm the roles of CH_4_ and CO in the AcOH synthesis process, we performed isotopic labeling tests over Pt-HPW-TiO_2_ with ^13^C labeled reactants. As shown in Figure 3a, when CH_4_ + ^13^CO was used, the ^13^C NMR spectrum contained three intensive narrow signals at 169.5, 182, and 187 ppm. The treatment by both labeled reagents, ^13^CH_4_ and ^13^CO, also showed the presence of a methyl group at 19 ppm (Figure 3b). Also, a very broad component in the 0–200 ppm range appeared.

According to the 2D ^1^H–^13^C CP-HETCOR data (Figure 3b), the signal at 169.5 ppm corresponds to carbon in the surface formate, H-^13^COO-, which is directly bonded with a proton, and the signal at δ_1H_ = 7.8 ppm is split by characteristic *J*^1^(^1^H–^13^C) coupling, ≈200 Hz. It should be mentioned that surface formate species are unstable and decompose readily to CO_2_ under atmospheric argon pressure (Figure 3a).^31^

The signals at δ_13C_ = 182 and 187 ppm can be assigned to C=O in the acetate species. They are close to protons of two methyl groups with corresponding chemical shifts (δ_1H_ = 1.5 ppm; δ_13C_ = 19 ppm and δ_1H_ = 2.1 ppm; δ_13C_ = 15 ppm) (Figure 3b,c). We suppose that these signals come from two different acetic groups, most likely bonded and coordinated to different sites. According to the literature, the signal at approximately 182 ppm could be due to the formation of surface acetate species strongly bonded to TiOH surface sites (CH_3_COO-Ti).^32^ The additional peak at 187 ppm may be related to the presence of a heteropolyacid unit nearby, with protonation of the O, which would cause a downfield shift of the carbon chemical shift in the acetyl group.

The exposure of the samples treated in methane and CO at the reaction temperature to water results in the complete disappearance of acetate groups in the ^1^H–^13^C CP/MAS NMR spectrum (Figure 3a). Only a broad signal in the 0–200 ppm range is observed, which can be attributed to carbon residuals coming mainly from methane. This suggests that treatment with water of Pt-HPW-TiO_2_ leads to the complete extraction of acetate from the nanocomposite. At the same time, NMR analysis of the liquid solution confirms the presence of AcOH with signals at δ = 20.8 and 178 ppm, which are ascribed to ^13^CH_3_^13^COOH and CH_3_^13^COOH when using labeled both CO and CH_4_ and only CO, respectively. That fact confirms that the methyl groups are derived from CH_4_, while the acyl groups of AcOH are produced from CO.

A possible mechanism of acetic acid synthesis from methane and CO could be based on the carbonylation of methanol, which involves intermediate generation of methanol from methane. To verify this route, we tested methane activation without CO, but no methanol was detected after water extraction (Figure S9, SI). It seems that methane activation requires the presence of CO. The optimal amount of CO in the reactor is in the range from 0.5 to 1 bar of CO, with a decrease in acetic acid production at a higher pressure. This suggests that methanol cannot be considered an intermediate in methane oxidative carbonylation over Pt-HPW-TiO_2_. It has to be noted that there is also no signal in NMR spectra related to methoxy species, which should be around 60 ppm.^33^ Thus, we could exclude the carbonylation of methanol, which had been used earlier for the synthesis of acetate over heteropolyacid catalysts.^34^

### Ex Situ and In Situ Characterizations

All the metal-HPW-TiO_2_ nanocomposite samples prepared by sequential impregnation of TiO_2_ support with aqueous solutions of HPW and metal salts contained 0.2 wt % of metal (Table S2, SI). According to STEM-HAADF and EDS mapping (Figures 2c,d and S3–S5, SI), the most active Pt-HPW-TiO_2_ (vide infra) demonstrates isolated Keggin-type HPW clusters over the surface of TiO_2_, with single-atom Pt attached to a heteropolyacid unit. Indeed, EDS mapping demonstrates a uniform distribution of Pt, in comparison with the arrangement of W in a unit containing 12 W atoms. In comparison with the impregnation of an aqueous solution of pure Pt salt over TiO_2_ leading to Pt nanoparticles, the method we propose leads to the generation of isolated Pt atoms due to the interaction of cationic Pt with the heteropolyacid and the subsequent stabilization of oxidized Pt by heteropolyacid anions. FTIR analysis of CO adsorption over freshly oxidized Pt-HPW-TiO_2_ demonstrates the presence of the main, single narrow peak at 2194 cm^–1^, indicating the presence of isolated oxidized Pt (Figure S10, SI).^35^ Heteropolyacid is soluble in water; however, it is still attached to TiO_2_ after calcination due to the ionic interaction of heteropolyacid anions possibly with Lewis acid sites on the TiO_2_ surface. Adsorption of Py over TiO_2_ before and after deposition of HPW, with subsequent calcination and washing, confirms a significant decrease in the number of Lewis acid sites, observed by the peak at 1446 cm^–1^, and an increase in the number of Brönsted acid sites, observed by the peak at 1536 cm^–1^ (Figure S11, SI). The supported stabilized heteropolyacids over TiO_2_ have been used earlier for different catalytic applications.^36,37^ Only niobium oxide (Nb_2_O_5_) demonstrates a performance similar to that of TiO_2_, which can be explained by the high acidity of both supports, resulting in interaction with heteropolyacid anions.

**Figure 4:**
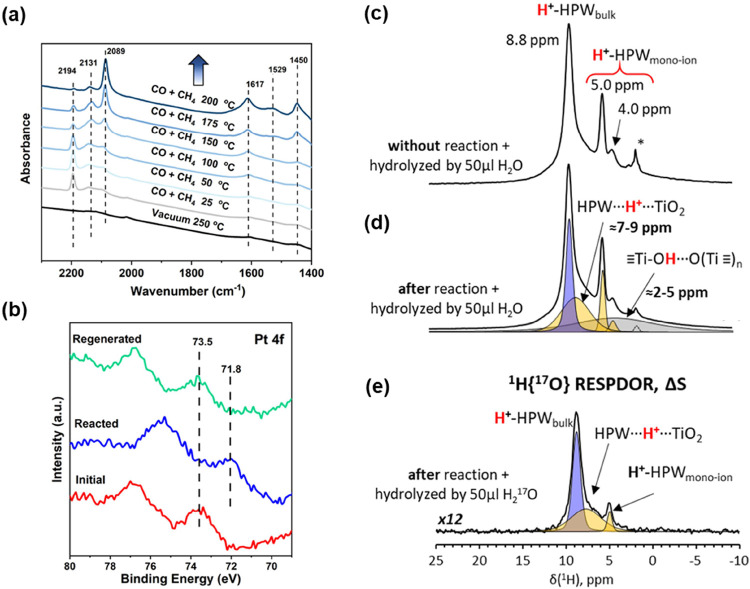
(a) In situ FTIR study of the reaction of CH_4_ with CO over Pt-HPW-TiO_2_ at different temperatures; (b) XPS analysis Pt 4f of initial Pt-HPW-TiO_2_, Pt-HPW-TiO_2_ after reaction, and Pt-HPW-TiO_2_ after regeneration; ^1^H HE/MAS NMR spectra of dehydrated Pt-HPW-TiO_2_ (c) original sample hydrolyzed by 50 μL H_2_O, (d) sample after the reaction and hydrolysis by 50 μL H_2_O, (e) ^1^H{^17^O} S-RESPDOR MAS NMR difference spectrum, Δ*S*, of dehydrated Pt-HPW-TiO_2_ after reaction and hydrolysis by 50 μL labeled H_2_^17^O with SR4_1_^2^ recoupling time of 100 μs.

The surface species on Pt-HPW-TiO_2_ under simulated reaction conditions have also been studied using in situ FTIR spectroscopy (Figure 4a). Initial CO adsorption over Pt-HPW-TiO_2_ demonstrates the presence of the peak at 2194 cm^–1^, which can be assigned to Pt^*n*+^–CO (with *n* = 2 or 4) in the form of PtO or PtO_2_ stabilized by heteropolyacid anions. The small broad peaks at 2131 and 2089 cm^–1^ can be assigned to the carbonyl of cationic Pt and reduced Pt, respectively.^35,38^

It is interesting to note that heating already at 100 °C in the presence of methane leads to the disappearance of the peak related to oxidized Pt, with an increase in the intensity of the peak at 2089 cm^–1^ of CO adsorbed over metallic Pt. Similar to our previous report,^12^ the variation of CO coverage did not affect the frequency of the peak at 2089 cm^–1^. This suggests that this peak can primarily be attributed to single-atom Pt, which also corresponds to the literature.^39^ STEM images also demonstrate the presence of Pt nanoparticles, which could have formed through the partial agglomeration of single-atom Pt (Figure S6, SI). Indeed, metallic Pt should be less stabilized by heteropolyacid on the surface of TiO_2_ compared to oxidized Pt. This process is also accompanied by the appearance of IR peaks at 1617, 1529, and 1450 cm^–1^, which could be assigned to the deformation vibration of water and the asymmetric (ν_s_) and symmetric (ν_as_) vibration of bidentate acetate ν(COO), respectively.^12^ The bands decrease in intensity after the treatment of the samples in vacuum (Figure S12, SI).

The evolution of the Pt state during the chemical looping process has been confirmed by XPS analysis (Figure 4b). The initial XPS 4f Pt spectrum reveals the presence of Pt in the oxidized state Pt^4+^, with peaks at 73.5 eV.^40^ The peak at 71.8 eV after the reaction with CO and CH_4_ and hydrolysis is attributed to Pt^0^. Reoxidation of the active sites after removing acetates was induced by air calcination at 250 °C and 1 bar of total pressure. The Pt sites have been reoxidized back to the initial state, as confirmed by XPS analysis.^41^

Thus, according to in situ FTIR and XPS, oxidized Pt plays a key role in the activation of methane to acetate with reduction to metallic Pt. Interestingly, no major sintering of Pt occurs during the reaction, and reduced Pt single-atom species characterized by an FTIR peak of adsorbed CO at 2089 cm^–1^ are mostly present after the reaction.

To identify the localization of the formed acetate after the reaction, we performed model experiments by treating TiO_2_ and the insoluble ammonia salt of HPW with glacial AcOH. After this treatment, we put the sample at 40 °C in a vacuum oven for 24 h and analyzed it by NMR. Figure S13, SI shows that the patterns for TiO_2_–AcOH were similar to those observed after methane coupling with CO, while no NMR peaks characteristic of acetates was detected over HPW-AcOH. It means that the formed acetate is stabilized over TiO_2_ rather than on HPW. This also explains the unique role of TiO_2_ in the formation of acetate. The stabilization of acetate over TiO_2_ has been observed earlier.^42^

The hydrolysis of acetates by water could proceed by C–O or Ti–O dissociation, with consumption of OH or H from water for AcOH synthesis. To clarify the actual route, we performed hydrolysis using H_2_^17^O. Liquid-phase analysis demonstrates only a single peak at 0 ppm related to H_2_^17^O and the absence of a peak around 250 ppm of labeled AcOH (Figure S14, SI). Solid-state ^1^H HE/MAS NMR analysis of the dehydrated samples shows that the fresh sample, without the reaction, contains several groups of characteristic signals (Figure 4c–e). Narrow signals correspond to free protons of either supported dehydrated HPW monoions (5.0 and 4.0 ppm)^43^ or bulk dehydrated HPW salt (8.8 ppm).^44^ The two broad components around 4 and 8 ppm can be attributed to H-bonded protons of (1) OH-groups of used amorphous TiO_2_^45^ and (2) acidic protons of HPW ions in contact with the TiO_2_ surface. Due to the high overlapping of the broad components, their positions can only be determined approximately. The reaction followed by hydrolysis with small amounts of liquid water did not affect the proton spectra of the samples (Figure 4b). The 1D difference ^1^H{^17^O} S*-*RESPDOR MAS NMR spectroscopy^46^ (Figures 4e and S15, SI) allows extracting the ^1^H NMR sub spectrum of protons bonded selectively with the ^17^O isotope after hydrolysis by labeled H_2_^17^O. According to these data, ^17^O easily enters into the bulk and isolated HPW ions (signals at 5.0 and 8.8 ppm). Moreover, the detection of broad components at ca. 8 ppm points to the appearance of ^17^O after hydrolysis in the HPW-TiO_2_ contact. At the same time, no detectable ^17^O-enrichment was observed for H-bonded titanol groups after the reaction and following hydrolysis. This allows us to conclude that hydrolysis may need a contribution from acidic HPW and occurs on TiO_2_ in direct contact with HPW.

### DFT Modeling

**Figure 5:**
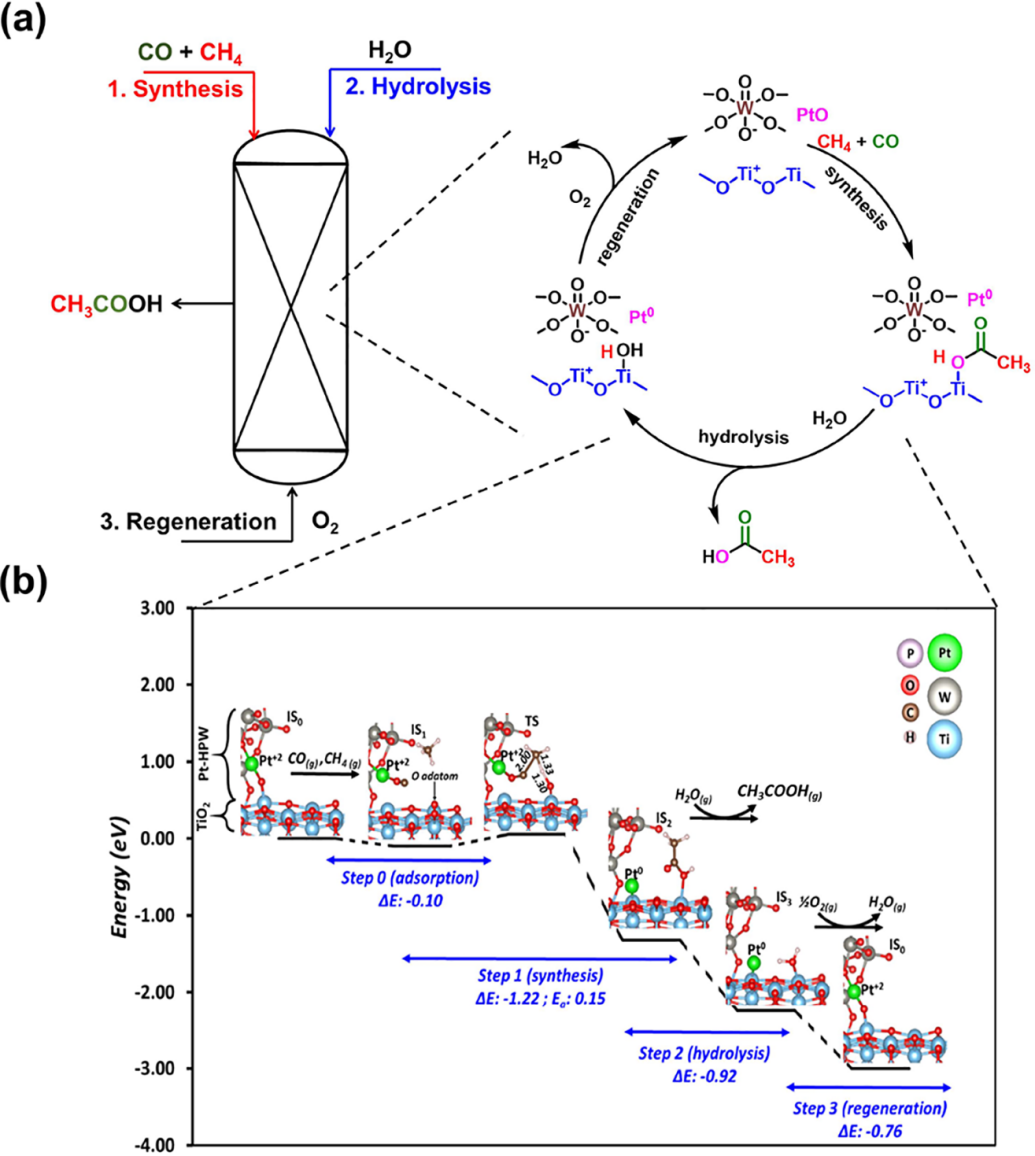
(a) Scheme of the looping process of methane coupling with CO for the synthesis of acetic acid including the steps of the synthesis, hydrolysis, and regeneration of Pt-HPW-TiO_2_. (b) Reaction energy diagram of acid acetic formation from CO/CH_4_with key surface intermediates and transition states ball and sticks models. Only the HPW/TiO_2_ interface was represented for clarity reasons. Pt computed oxidation states are presented for each intermediate.

Density functional theory calculations were employed to further probe the reaction mechanisms, details of which are provided in the Supporting Information. Here, we simulated the mechanism of AcOH formation from methane (CH_4_) and carbon monoxide (CO). The reaction process was divided into three steps, as shown in Figures 5, S16, and S17, SI. The computed Pt oxidation states were identified for each intermediate derived from Bader charge analysis. In the initial state, the Keggin anion is preferentially adsorbed on TiO_2_, with a Pt atom connected to the surface via an O atom, while CO and CH_4_ are in the gas phase. While the two molecules approach the sample, the O atom, previously linking Pt to TiO_2_, shifts upward slightly as a surface adatom, hence allowing CO to bind the vacant Pt atom, with CH_4_ physisorbed nearby (step 1). Then, in the concerted mechanism, we identify a series of concerted reactions of C–H breaking in the CH_4_ molecule, C–C bonding between CH_4_ and CO, C–O bonding between CO and the O adatom at the surface, and O–H bonding between the O adatom and the H resulting from C–H scission in CH_4_, leading to the formation of AcOH. The AcOH adsorbate is then removed from the surface via hydrolysis (step 2). Finally, the surface is regenerated in the last step by partial oxidation (step 3). Let us mention that although the Pt atom is initially shared between HPW and TiO_2_, strongly attached by two O atoms (step 1), during subsequent AcOH production, the Pt atom is expelled from the HPW macrostructure and directly binds a neighboring Ti atom at the surface. This explains the partial segregation of Pt into metal nanoparticles after the reaction (Figure S3, SI), with subsequent regeneration. This finding from our computational model is aligned with experimental observations, reporting Pt reduction during the reaction. The model presented for Pt in oxidation state 2+ should result in the production of one molecule of AcOH per Pt atom. However, our results show that the amount of AcOH corresponds to 1.5 of Pt, which could be assigned to the oxidation state of Pt^4+^, corresponding to PtO_2_. As appears in Figure 5, each step is exothermic, with considerable reaction energy values (Δ*E*), even reaching −1.22 eV for AcOH formation in step 1. Thus, the reaction process is highly favorable on the thermodynamic aspect, as confirmed by a global reaction energy of −3.0 eV in the gas phase. Only focusing on the main reaction step (AcOH formation), the kinetics are also clearly advantageous, with a very low barrier of 0.15 eV for step 1. All those results attest to the feasibility of the proposed mechanism, demonstrating the outstanding capabilities of our ternary-phase sample, Pt-HPW-TiO_2_. The total reaction can be described by the equation: CH_4_ + CO + 1/2O_2_ = CH_3_COOH.

In comparison with our previous study on the photocatalytic carbonylation of methane in the presence of water, where methane activation is performed by photogenerated OH radicals,^12^ the activation of methane in the thermochemical route occurs by oxidized Pt species, which simultaneously undergo reduction. Different to photocatalytic methane carbonylation^12^ to acetic acid, reoxidation of Pt reduced species cannot be achieved by water, which under UV light generates a large concentration of OH radicals. Instead, exposure of the reduced Pt-HPW-TiO_2_ nanocomposite to air at higher temperatures is required to close the chemical loop.

The importance of the presence of three components such as Pt, HPW, and TiO_2_ for the synthesis of AcOH from methane and CO can be assigned to the individual function of each of them. Oxidized PtO_*x*_ is required to activate methane by dissociation of the C–H bond with simultaneous coupling with CO to form acetate. The role of the heteropolyacid according to the mechanism of the reaction is in the stabilization of single-atom Pt, providing oxygen transfer to TiO_2_ for stabilization of acetate, and in providing acid sites for hydrolysis of acetate. The role of TiO_2_ is in sharing oxygen with Pt and capturing acetate to form Ti–O–Ac species for subsequent hydrolysis.

This chemical looping process offers the opportunity to achieve a high concentration of acetic acid with high selectivity, making it an interesting approach compared to the literature (Figure S18 and Table S1, SI). Traditional routes for the oxidative carbonylation of methane to acetic acid over Cu-, Ir-, or Rh-containing catalysts require the intermediate oxidation of methane to methanol, followed by the subsequent carbonylation of methanol to acetic acid. This process results in the generation of methanol as a side product and limits the concentration of the formed acetic acid.

## Conclusions

We demonstrated a chemical looping strategy for the synthesis of AcOH by the oxidative carbonylation of methane. The sample for the reaction consists of TiO_2_ and PtO_*x*_ anchored to phosphotungstic heteropolyacid attached to TiO_2_. The sample enables the selective and stable synthesis of AcOH in the amount corresponding to 1.5 atoms of Pt, with a liquid-phase selectivity close to 100% and yielding the AcOH solution with a concentration up to 1.1 wt %. A combination of isotope labeling and in situ characterization suggests that CH_4_ is activated by oxidized Pt and reacts with a CO molecule yielding an acetyl group over the TiO_2_ surface. The acetyl group is then hydrolyzed by water at the interface of TiO_2_ and HPW to form an AcOH solution, and the sample should be calcined afterward to regenerate PtO_*x*_ sites for the new reaction cycle. The route proposed in this work for direct acetic acid synthesis from methane results in selective synthesis with the potential to achieve high acetic acid concentrations at high selectivity.

## Supporting Information Available

Gas production, regeneration, HAADF-STEM images materials, optimization of the reaction of acetic acid synthesis, FTIR analysis of CO and Py adsorption, NMR analysis, DFT modeling of energy and transition state, comparison with the literature, and chemical analysis (PDF)
