# Supplementary material for: Stoichiometric Selective Carbonylation of Methane to Acetic Acid by Chemical Looping
Source: ACS Catal. 2025 Feb 5;15(4):3116–25. doi: 10.1021/acscatal.4c07095 (PMC11851441; doi:10.1021/acscatal.4c07095)
Supplement: Supplementary file 1 — cs4c07095_si_001.pdf [file cs4c07095_si_001.pdf]

Supporting Information

Stoichiometric selective carbonylation of methane to acetic acid by chemical looping

Yinghao Wang[a,#] Chunyang Dong[a,b,#] Mariya Shamzhy[c], Maya Marinova,[d] Zhengxiao Guo[b*], Yury G. Kolyagin[a*], Jeremie Zaffran[e*], Andrei Khodakov[a*] and Vitaly V. Ordomsky[a*]

[a] *UCCS–Unité de Catalyse et Chimie du Solide, Université de Lille, CNRS, Centrale Lille, ENSCL, Université d'Artois, UMR 8181, Lille, France.*

[b] *Department of Chemistry, The University of Hong Kong, Hong Kong, China.*

[c] *Department of Physical and Macromolecular Chemistry, Faculty of Science, Charles University, Hlavova 2030/8, 12843 Prague, Czech Republic*

[d] *Institut Michel-Eugène Chevreul, 59655 Villeneuve-d'Ascq, France*

[e] *Eco-Efficient Products and Processes Laboratory (E2P2L), IRL 3464 CNRS-Syensqo, 3966 Jin Du Road, Xin Zhuang Ind. Zone, 201108 Shanghai, China;*

[#] equal contribution

Email addresses: zxguo@hku.hk; Yury.Kolyagin@univ-lille.fr; Jeremie.Zaffran@cnrs.fr; Andrei.Khodakov@univ-lille.fr; Vitaly.Ordomsky@univ-lille.fr

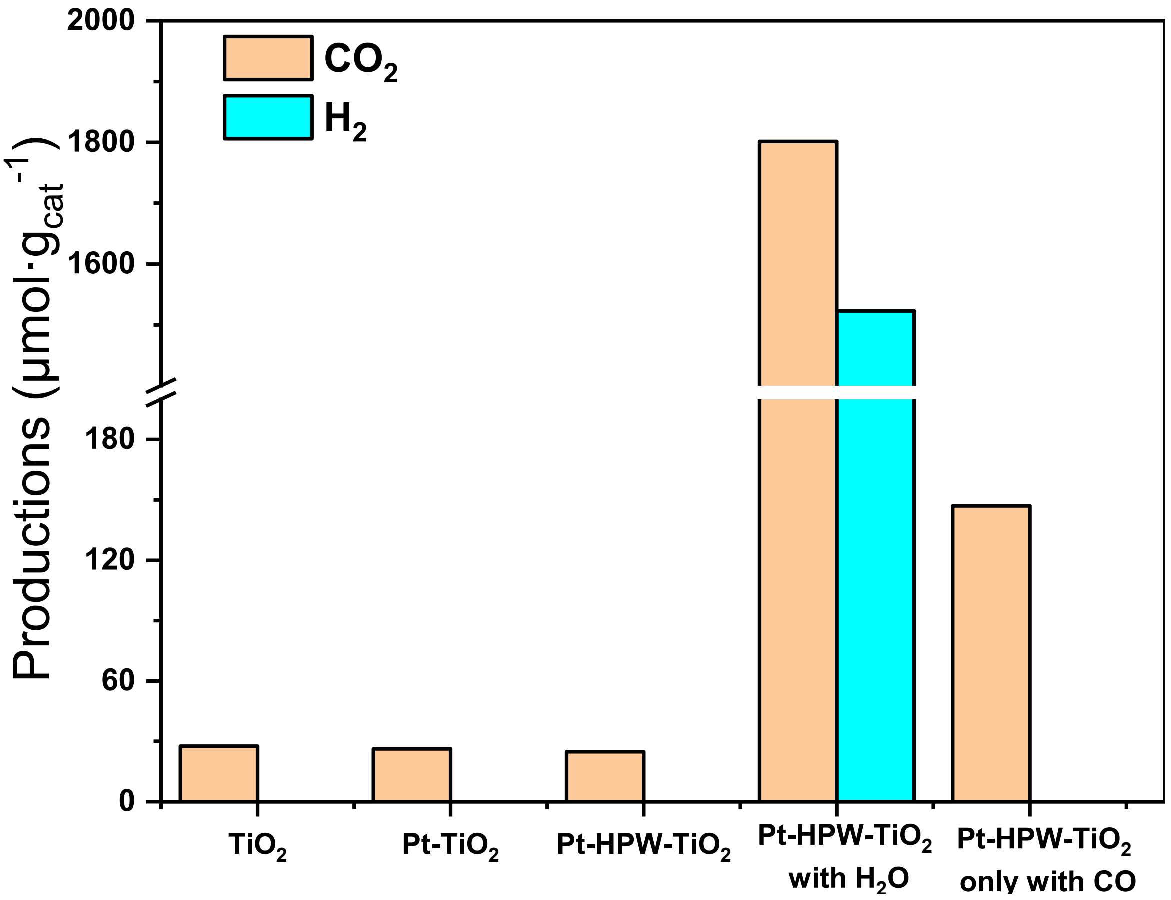

Figure S1. *The gas product over TiO_2, Pt-TiO_2, Pt-HPW-TiO_2, Pt-HPW-TiO_2 in H_2O with CH_4 and CO and Pt-HPW-TiO_2 only with CO after reaction. General reaction conditions: 50 mg samples, 15 bar CH_4 (sample only with CO will be added 15 bar N_2), 1 bar CO, 175 ^oC, 2 h reaction time. In the case of test with water, 1 g of water has been added in the reactor.*

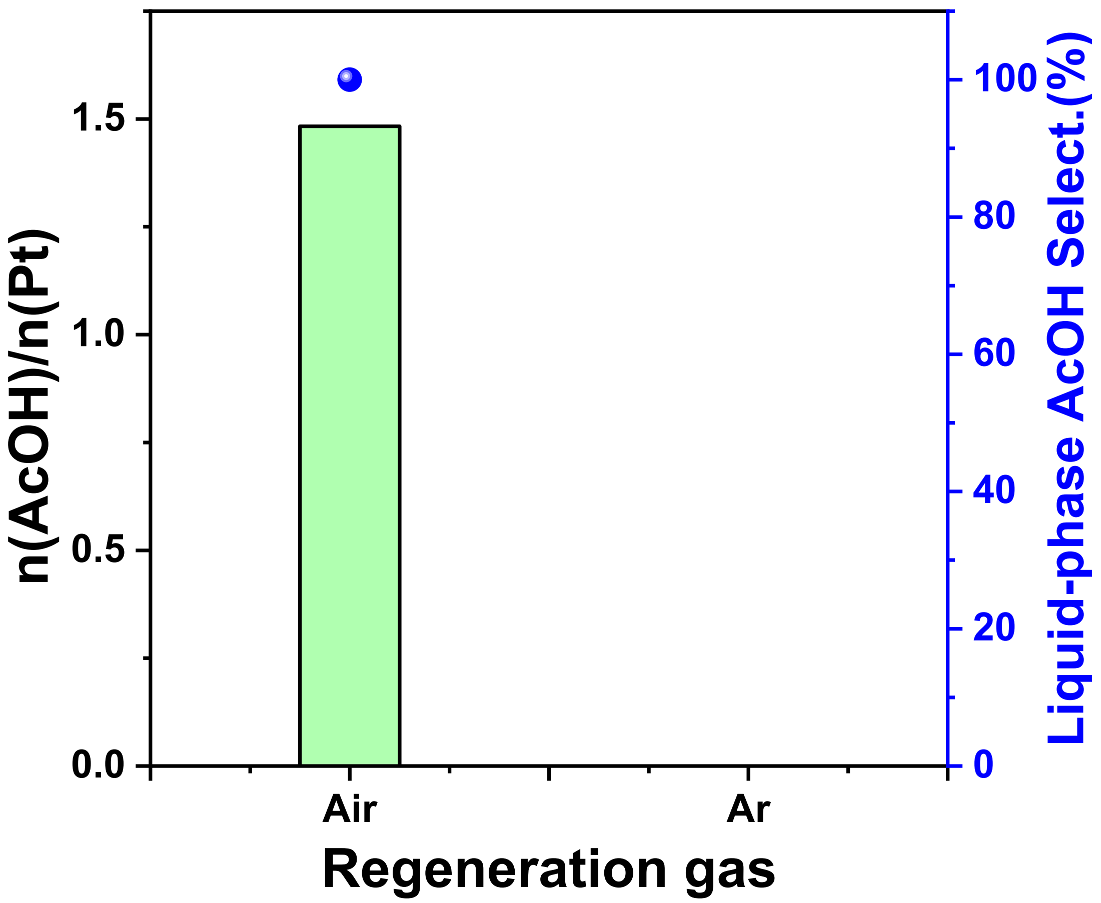

Figure S2. *Comparison of the AcOH synthesis performance of Pt-HPW-TiO_2 using either air or Ar as the regeneration atmosphere.*

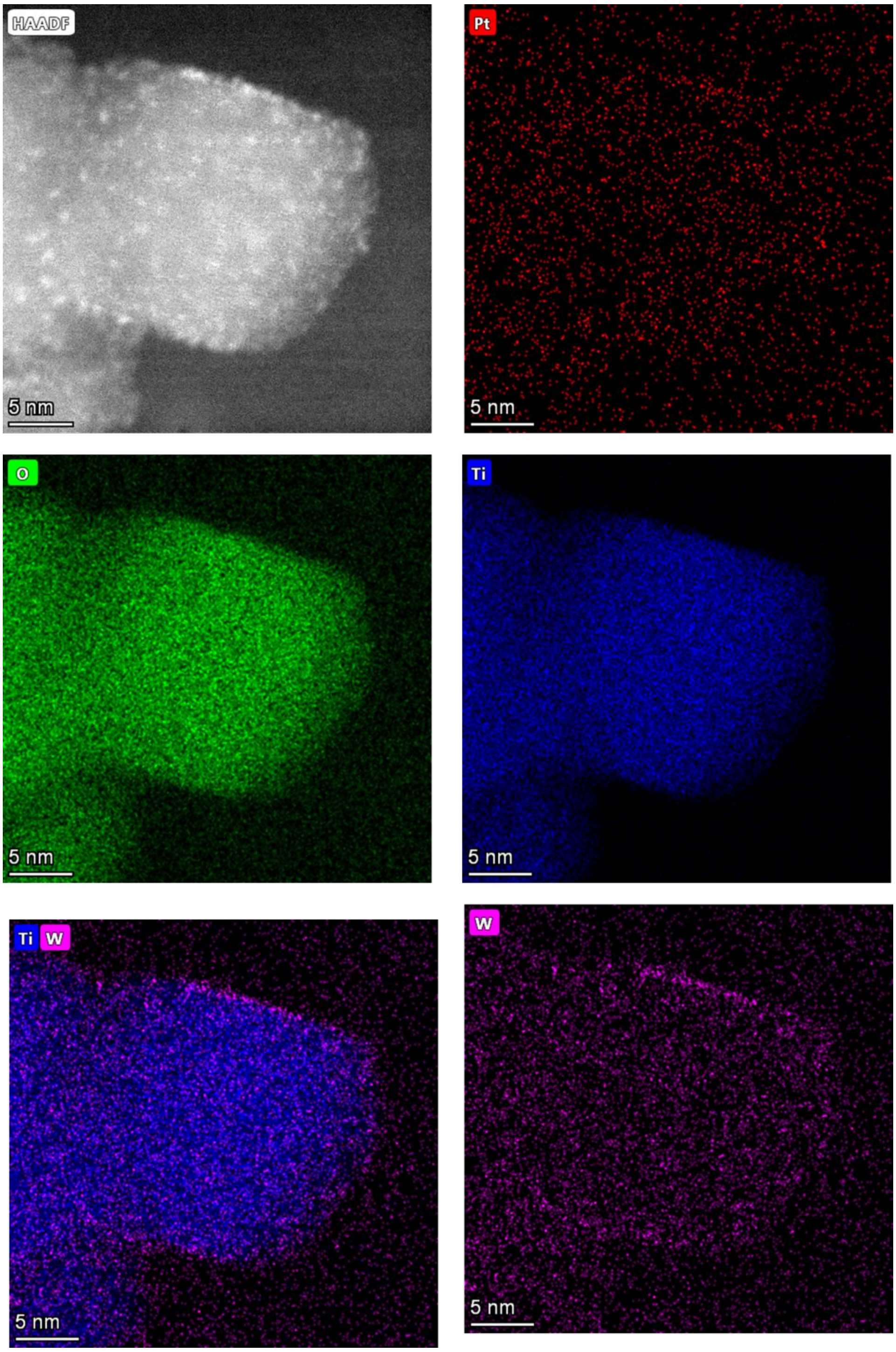

Figure S3. *HAADF-STEM and corresponding EDS-mapping images of Pt-HPW-TiO$_2$ before reaction.*

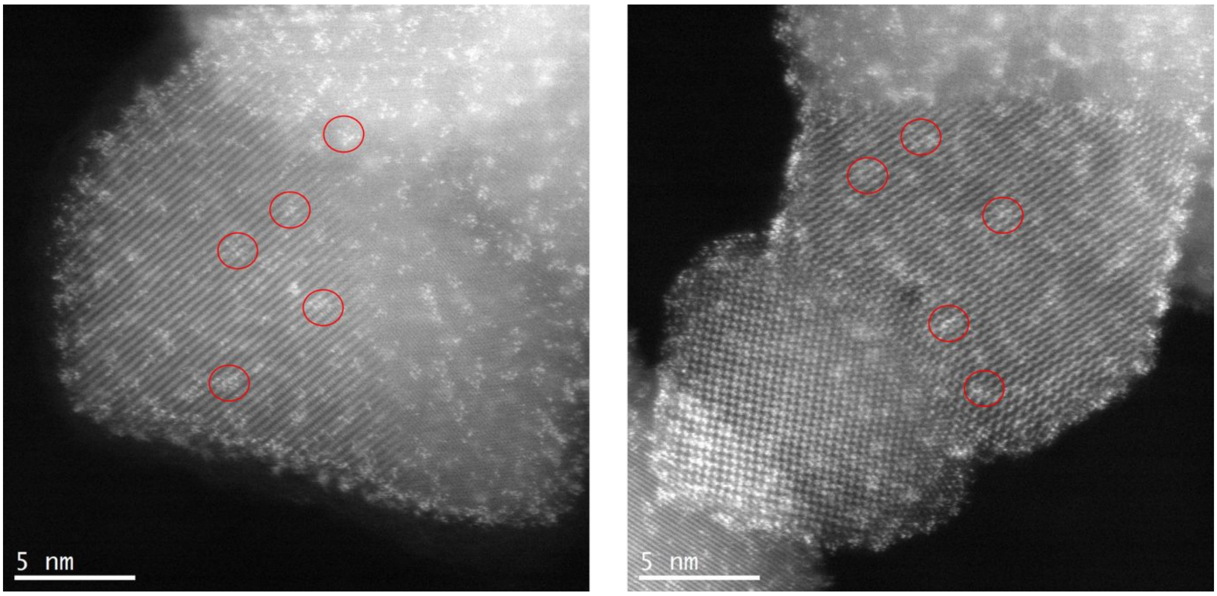

Figure S4. *HAADF-STEM images of Pt-HPW-TiO_2 before reaction with demonstration of heteropolyacid units (circle)*

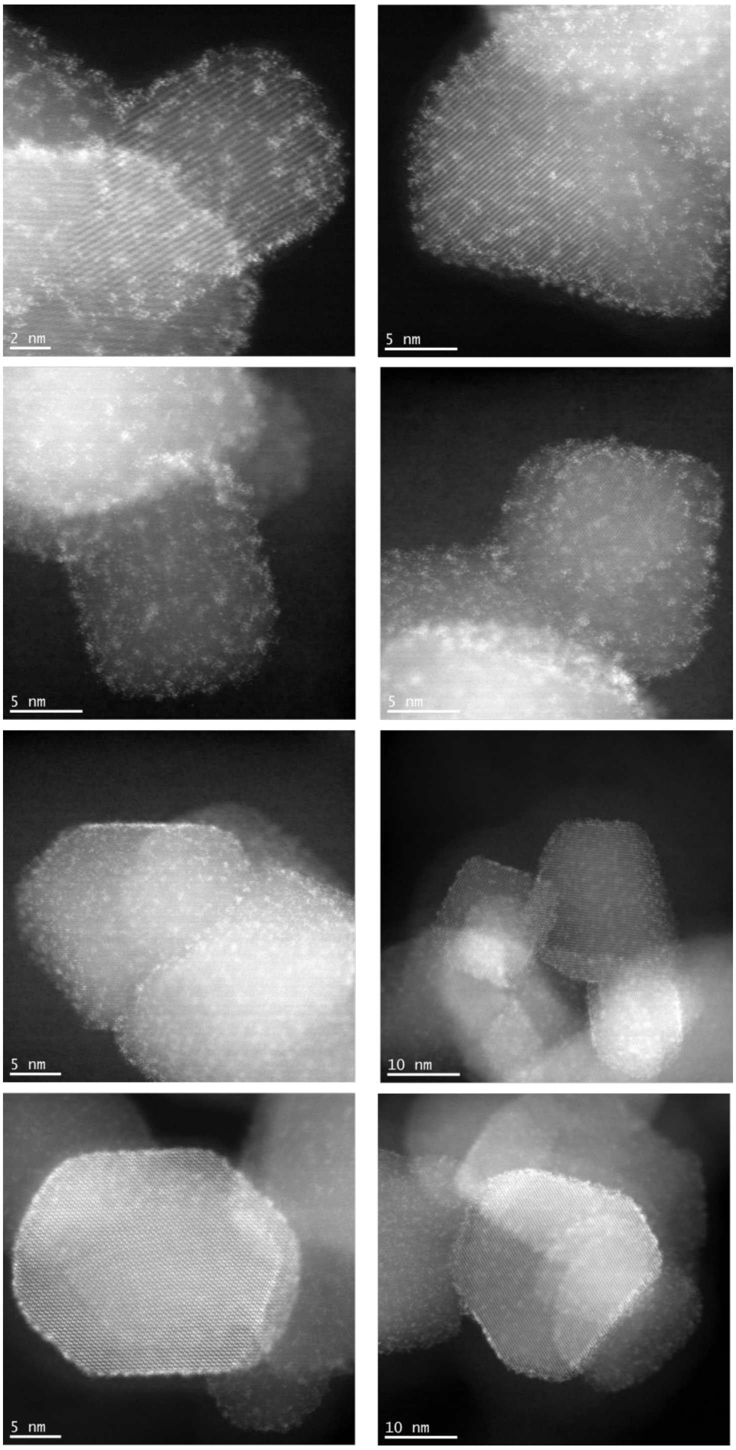

Figure S5. *HAADF-STEM images of Pt-HPW-TiO$_2$ before reaction*

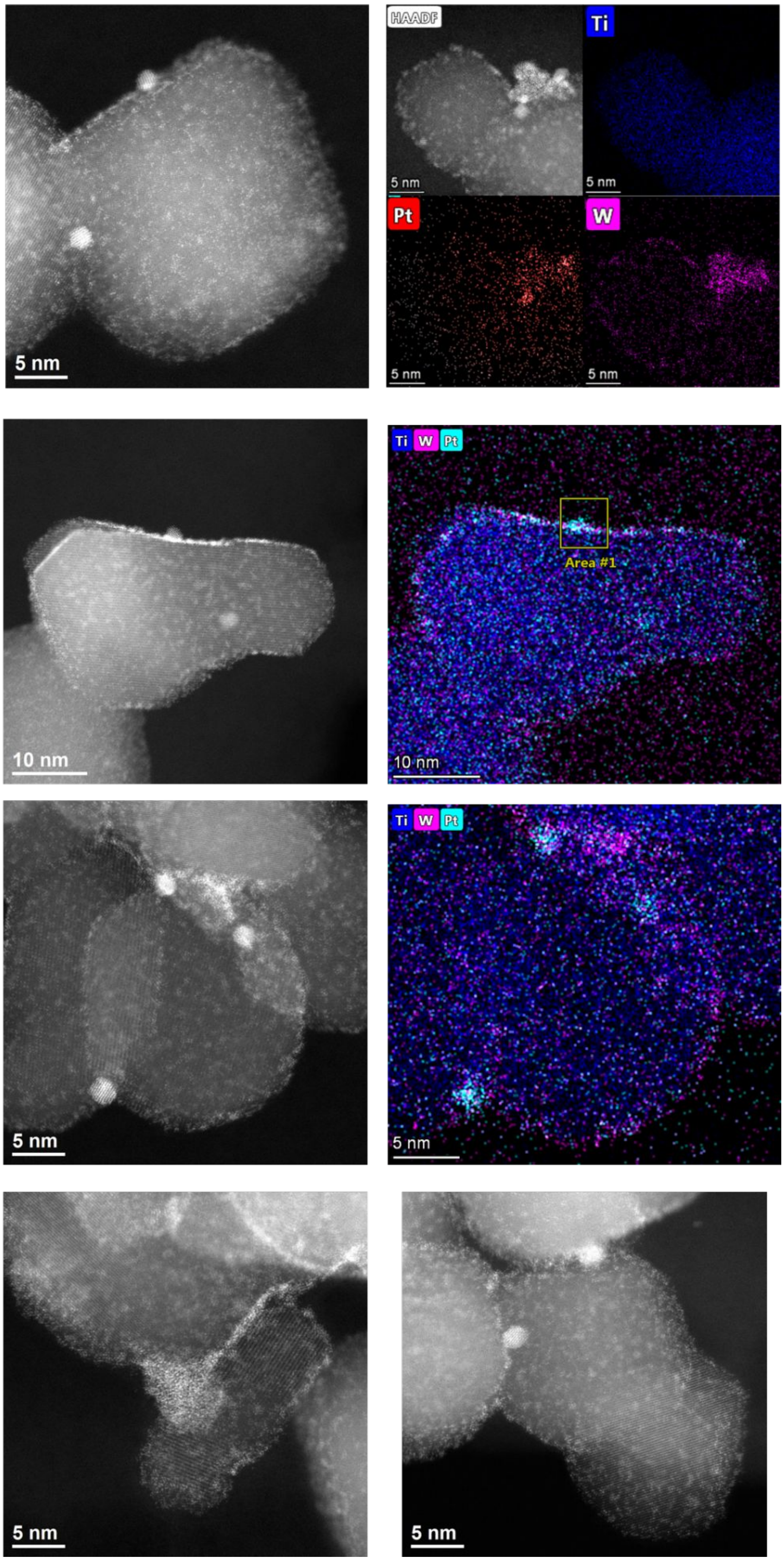

Figure S6. *HAADF-STEM and corresponding EDS-mapping images of Pt-HPW-TiO_2 after reaction.*

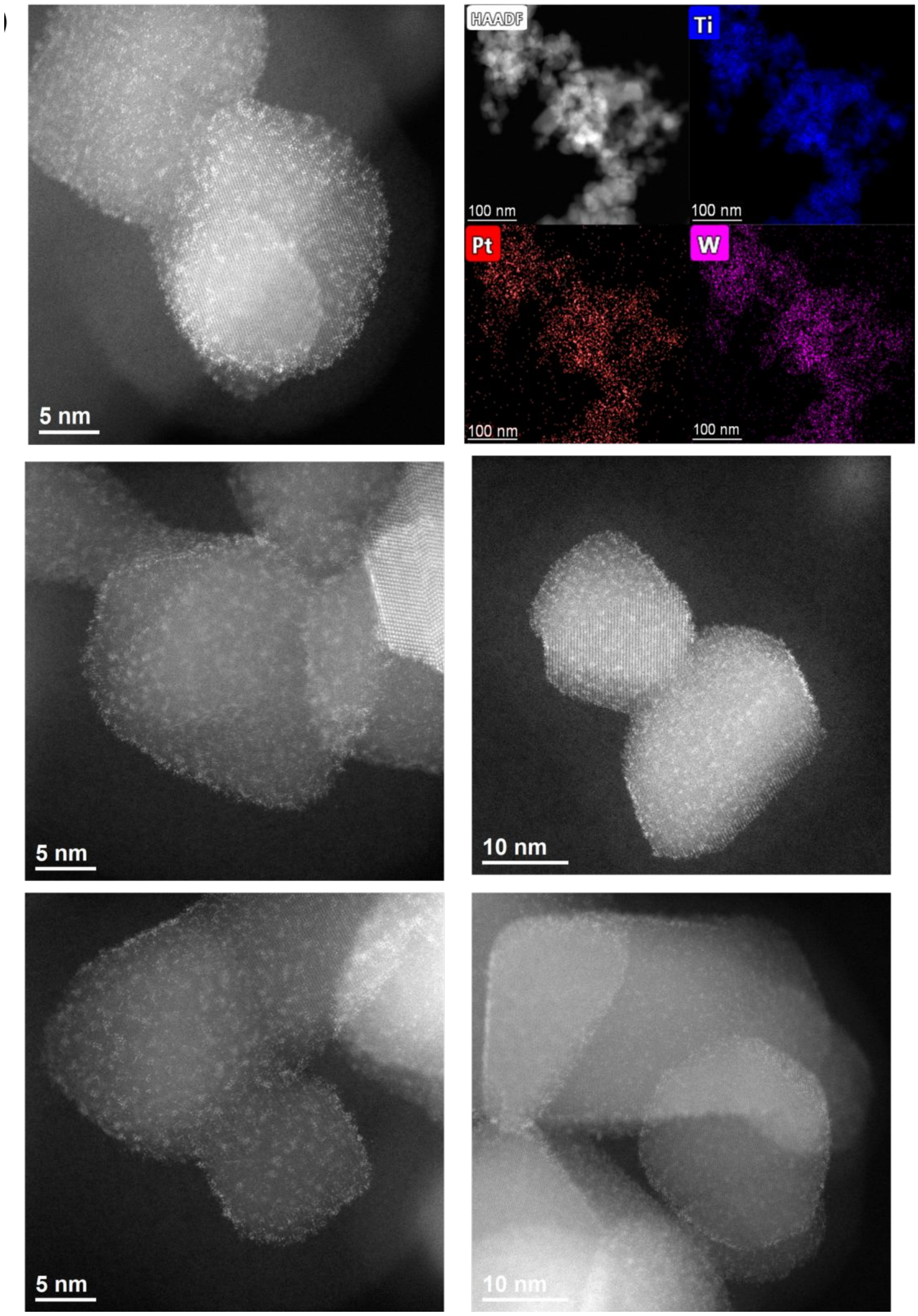

Figure S7. *HAADF-STEM and corresponding EDS-mapping images of Pt-HPW-TiO_2 after regeneration.*

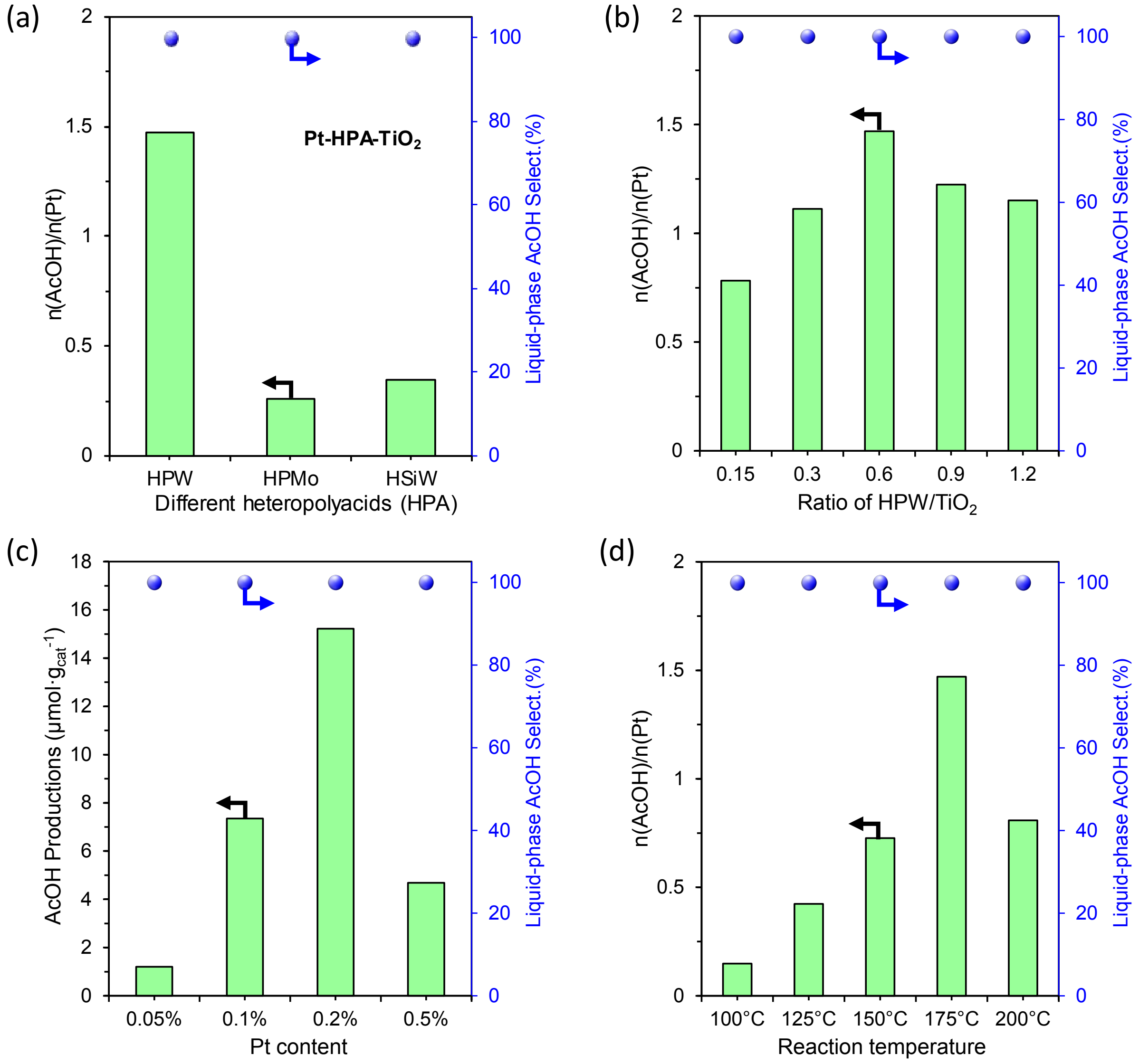

Figure S8. *The optimization of the reaction temperature and of material characteristics for acetic acid synthesis on Pt-HPW-TiO_2. General reaction conditions: 50 mg samples, 15 bar CH_4, 1 bar CO, 2 h reaction time.*

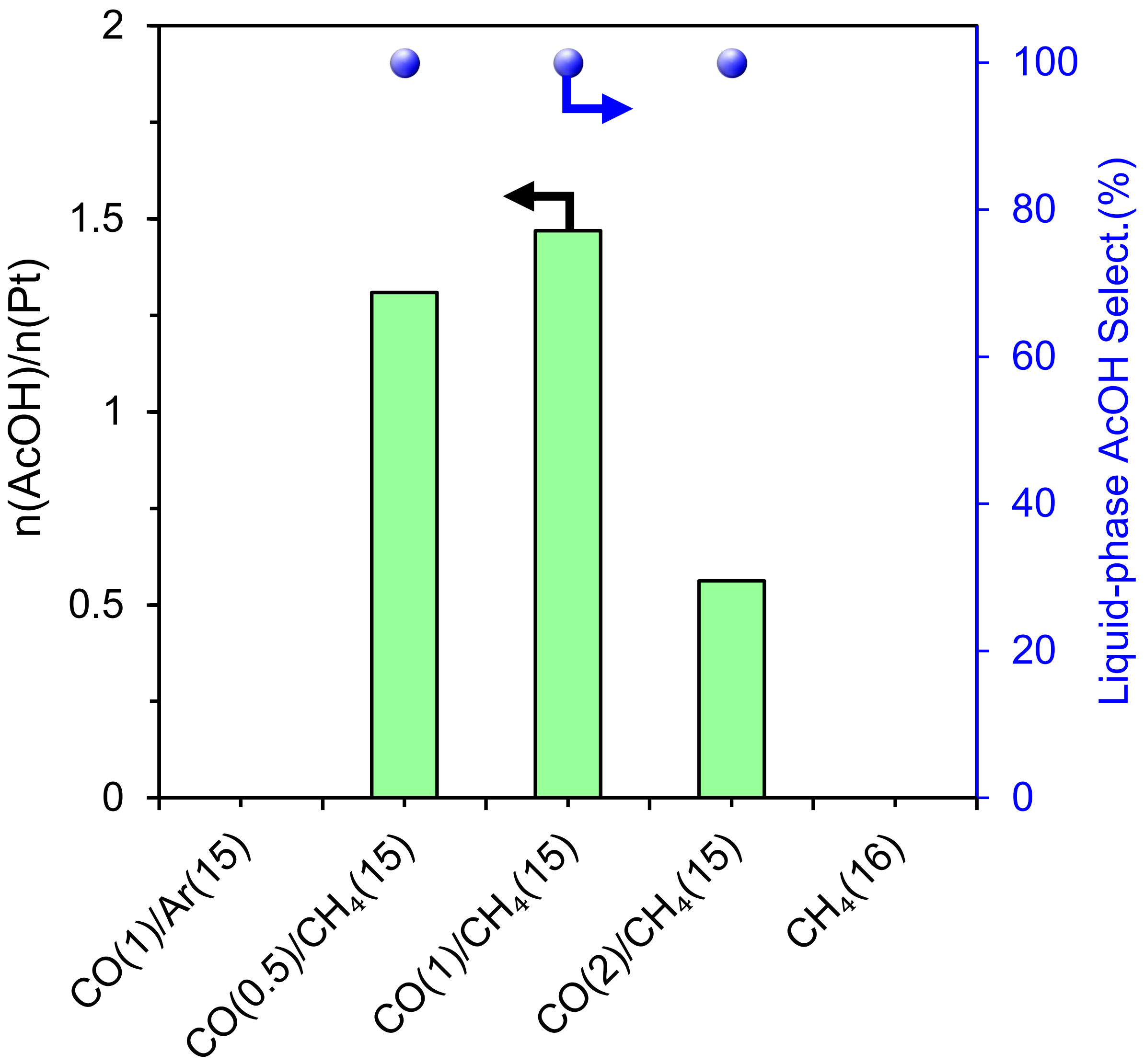

Figure S9. *The reference tests of different gas ratios on Pt-HPW-TiO$_2$. General reaction conditions: 50 mg samples, 175 ^oC, 2 h reaction time.*

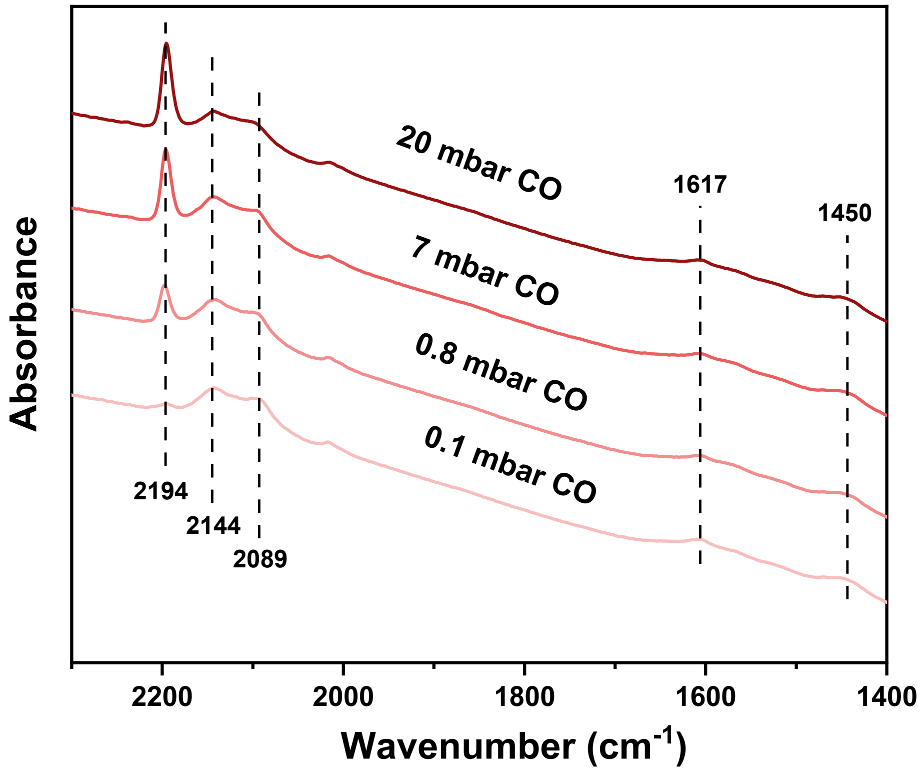

Figure S10. *FTIR analysis of CO adsorption with subsequent desorption over prepared Pt-HPW-TiO_2*

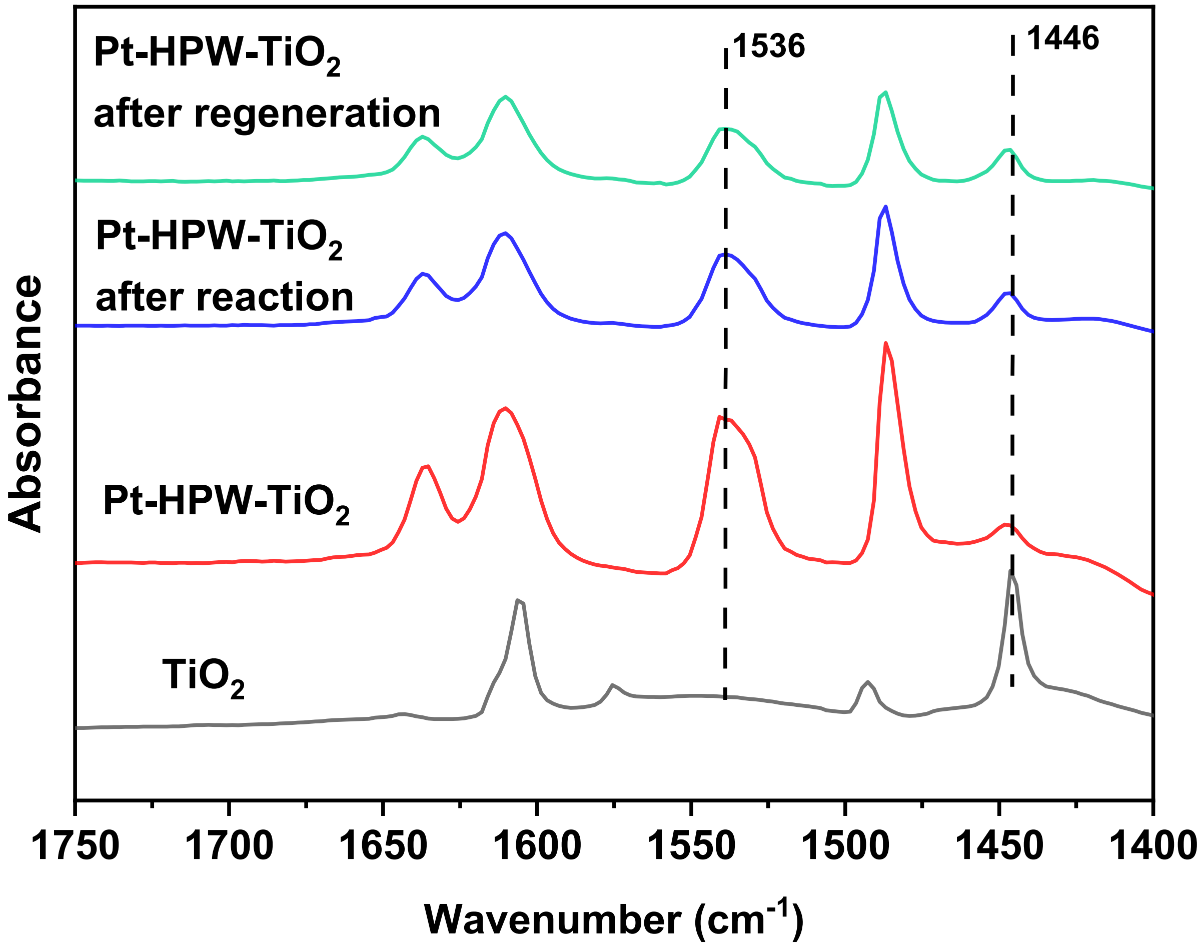

Figure S11. *FTIR analysis of Py adsorption over* TiO_2*, Pt-HPW-*TiO_2 *before and after reaction and after regeneration.*

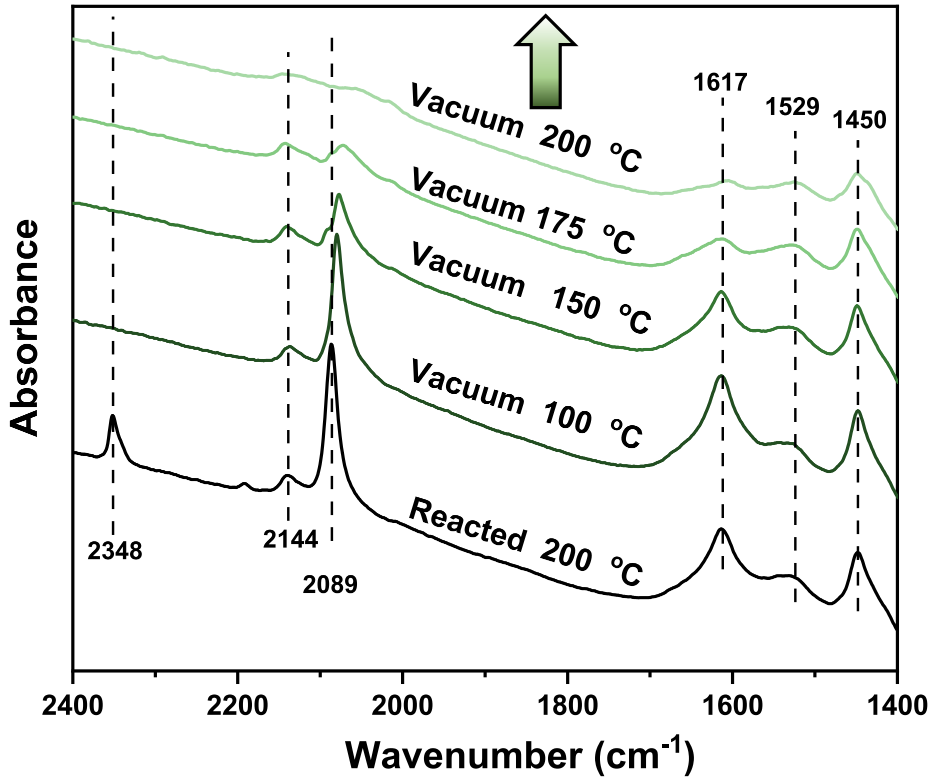

Figure S12. *FTIR-monitored thermal desorption of acetic acid on Pt-HPW-TiO$_2$.*

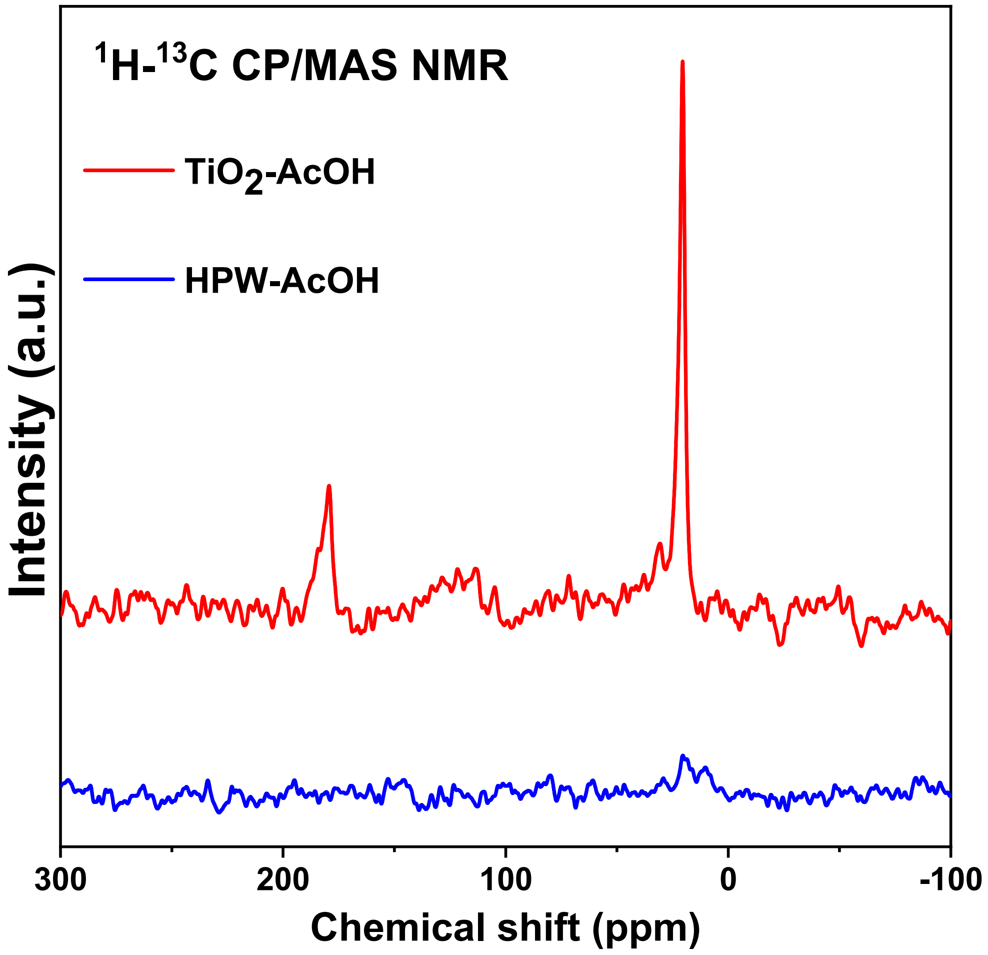

Figure S13. *^{1}H-^{13}C CP/MAS NMR spectra of treating TiO_2 and insoluble ammonia salt of HPW with glacial AcOH.*

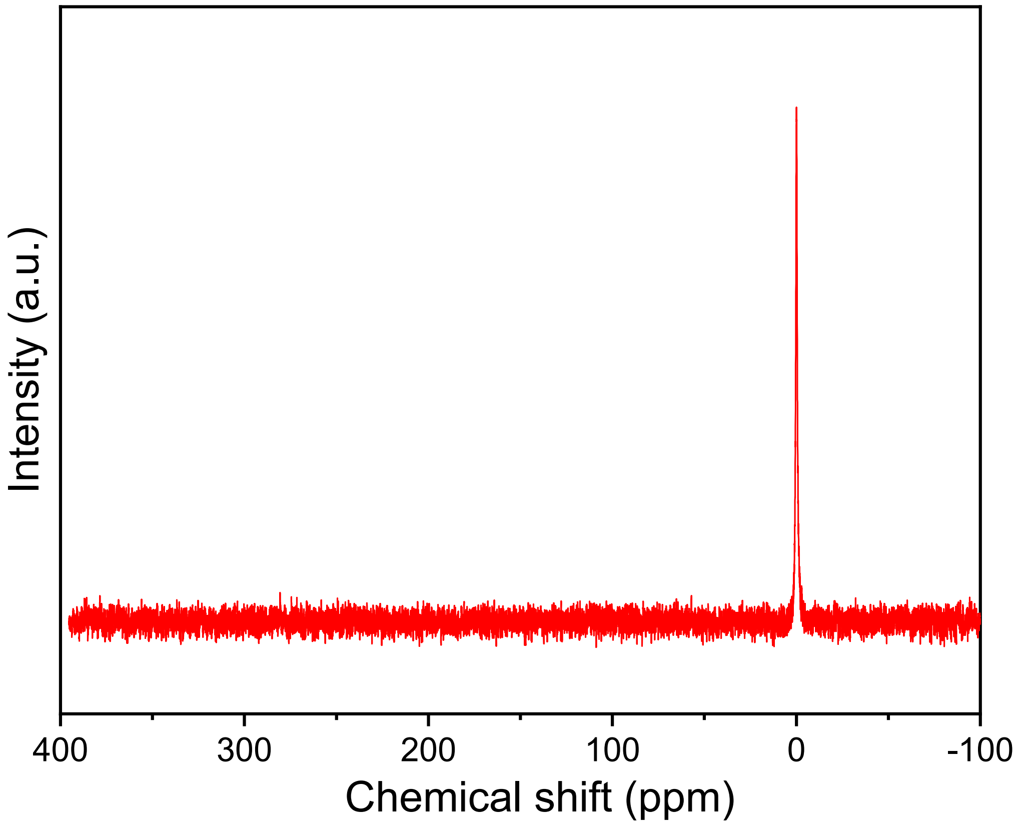

Figure S14. *^{17}O NMR analysis of liquid solution after hydrolysis using $H_2{}^{17}O$.*

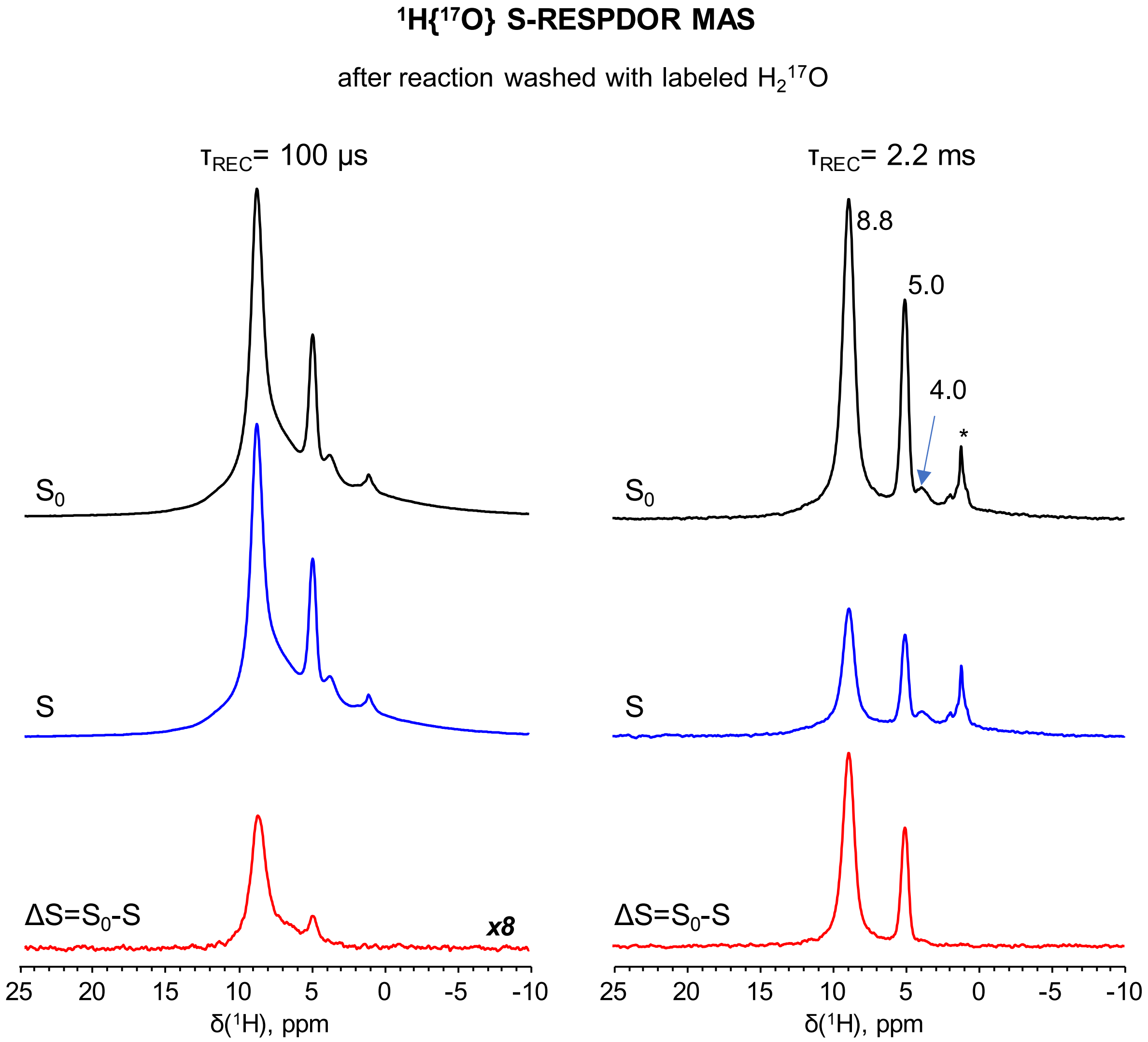

Figure S15. *^1H-{^{17}O} S-RESPDOR MAS NMR spectra without (S_0) and with (S) ^{17}O saturation pulse and corresponding difference ($\Delta S=S_0-S$) of dehydrated Pt-HPW-TiO_2 material after reaction and hydrolysis by 50µl labeled $H_2{}^{17}O$ with $SR4_1^2$ recoupling time of 100 µs and 2.2 ms.*

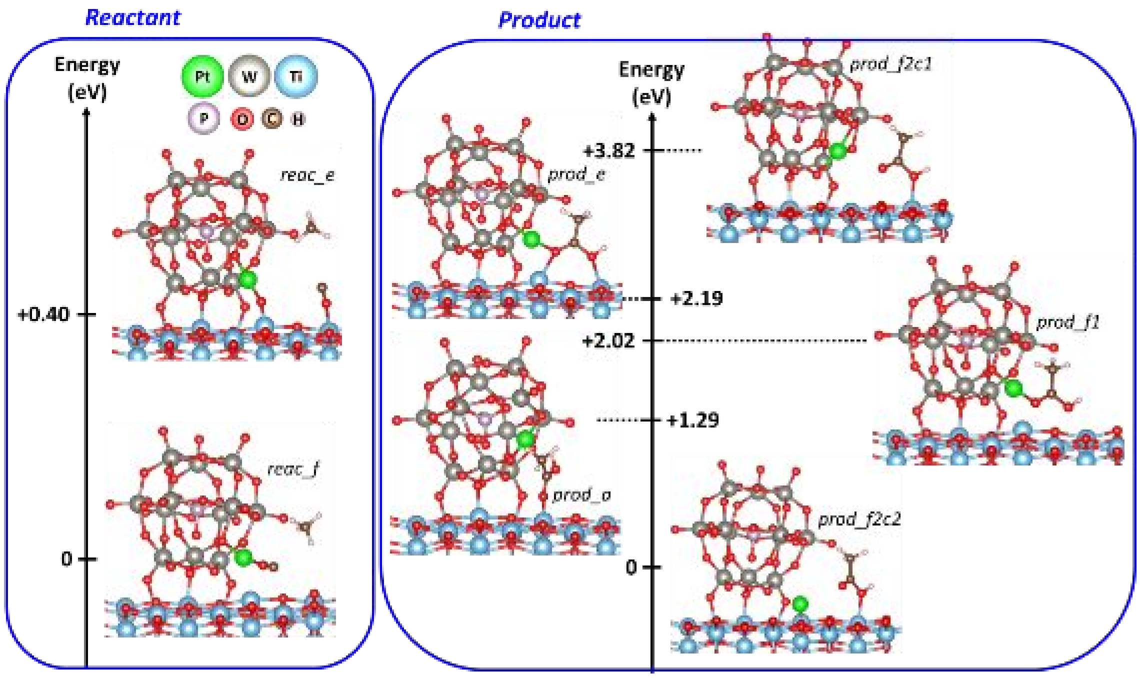

Figure S16. *Configuration screening for the reactant and the product states, with the relative energies of the various adsorption modes at Pt-HPW-TiO_2.*

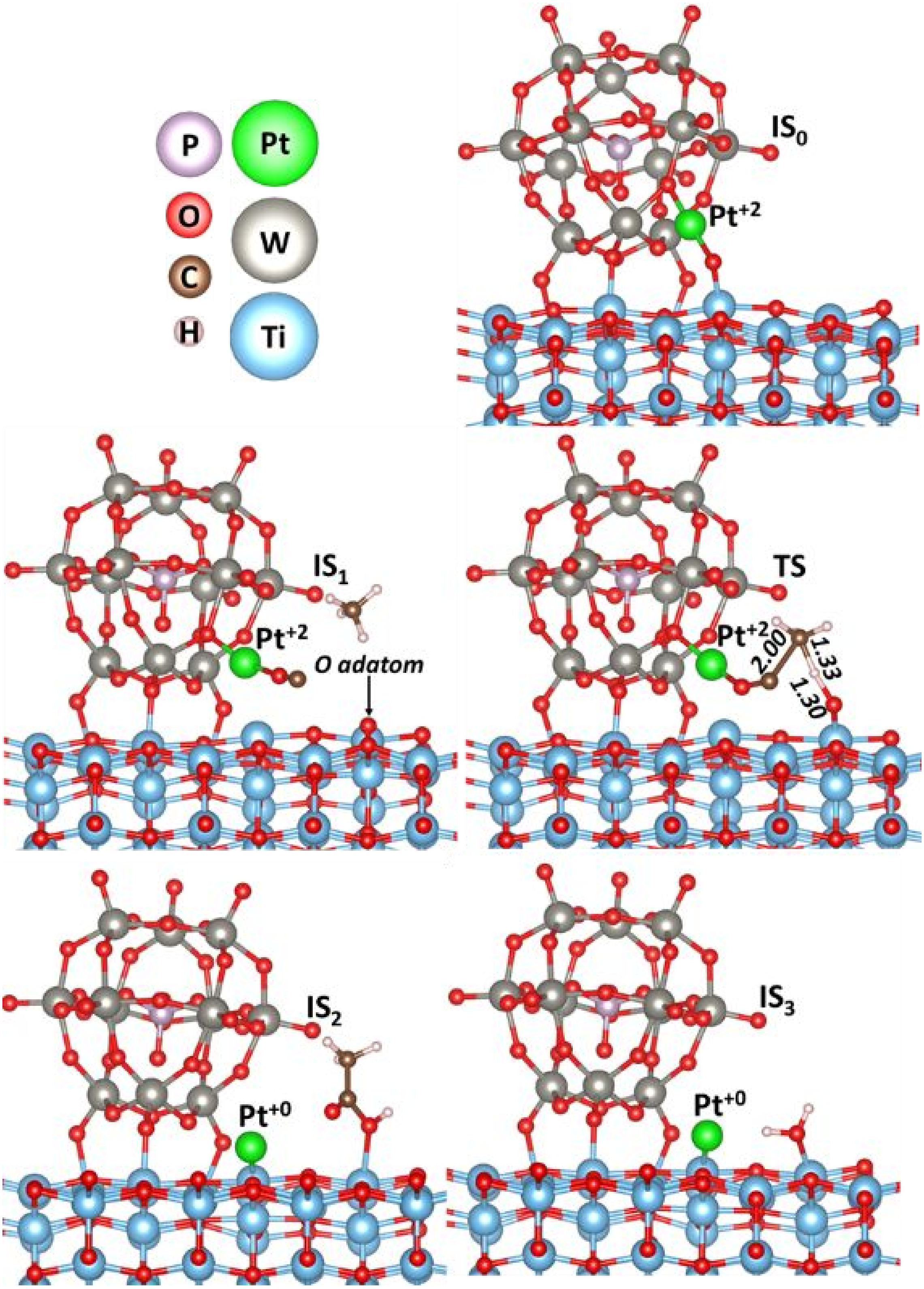

Figure S17. *Reaction intermediate and transition state balls & sticks model related to acetic acid formation from CO and water (see reaction path in Fig. 5 of the manuscript).*

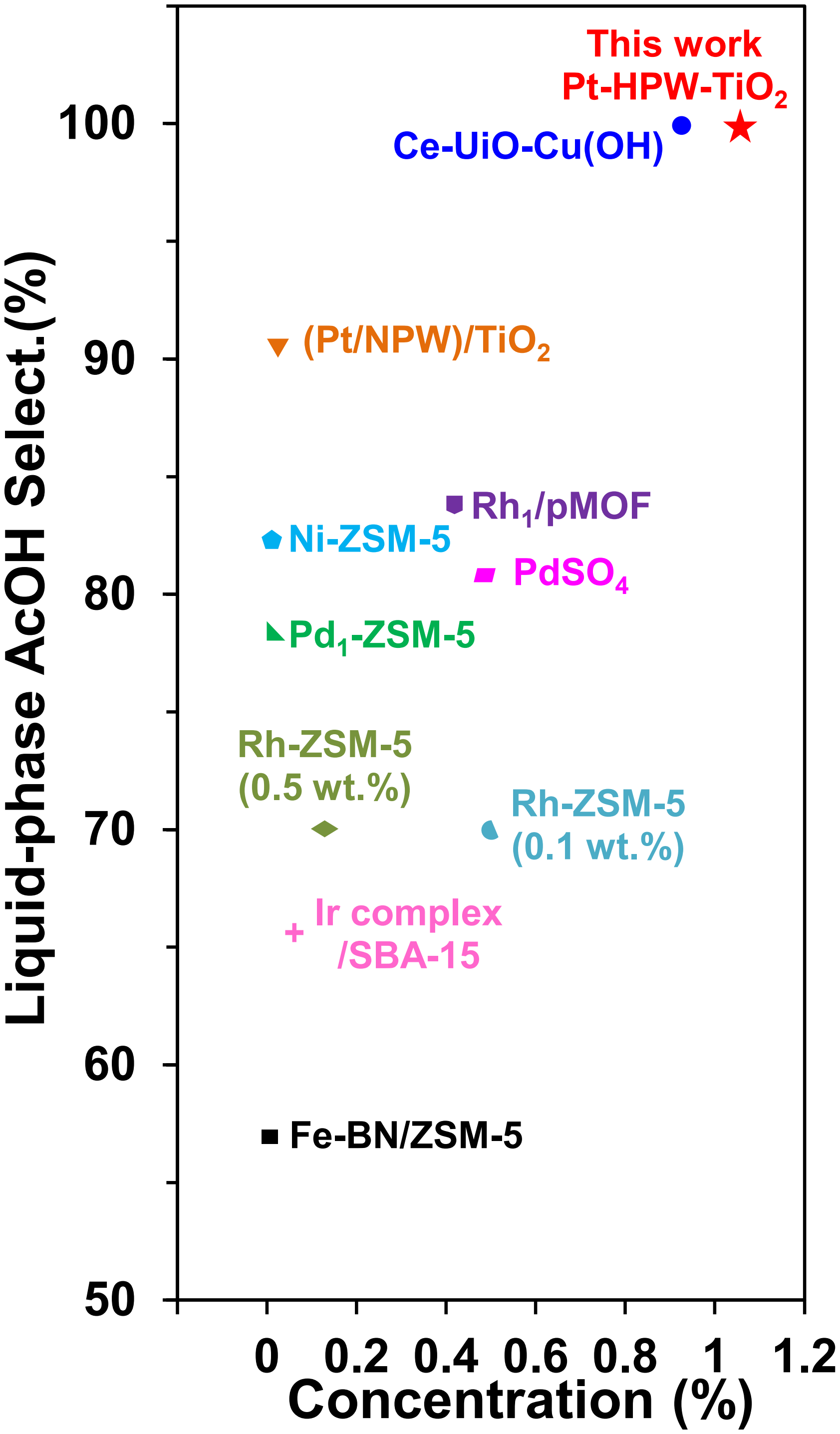

Figure S18. *Literature comparisons of the AcOH concentration and AcOH selectivity in liquid-phase. References are as follows: $PdSO_4$ (1), Rh-ZSM-5 (0.5 wt.%) (3), Rh-ZSM-5 (0.1 wt.%) (4), Fe-BN/ZSM-5 (6), Ce-UiO-Cu(OH) (8), (Pt/NPW)/TiO_2 (9), Ir complex /SBA-15 (10), Rh_1/pMOF (11), Pd_1-ZSM-5 (12), Ni-ZSM-5 (13).*

Table S1. *Comparison of the existing conversion routes for the synthesis of acetic acid from methane*

Name	Reaction conditions	AcOH Conc. (wt. %)	AcOH Conc. (mmol/L)	Liquid-phase AcOH Select. (%)	Ref.
$PdSO_4$	20 mM $PdSO_4$, 96% H_2SO_4, 180 ℃, 7 h	0.49	82	67.9	1
Cu-mordenite	1.5 g samples, 50 mL/min O_2, 10 mL/min CH_4, 200 ℃, 0.5 h	-	-	48.7	2
Rh-ZSM-5 (0.5 wt.%)	20 mg samples, 2 bar O_2, 5 bar CO, 20 bar CH_4, 20 mL H_2O, 150 ℃, 3 h	0.13	21.3	70.1	3
Rh-ZSM-5 (0.1 wt.%)	28 mg samples, 8 bar O_2, 10 bar CO, 50 bar CH_4, 10 mL H_2O, 150 ℃, 12 h	0.50	84.0	70.1	4
Au-ZSM-5	100 mg samples, 1 bar O_2, 2.5 bar CO, 20.7 bar CH_4, 15 mL H_2O, 240 ℃, 4 h	0.003	0.5	12.7	5
Fe-BN/ZSM-5	20 mg samples, 5 bar CO, 25 bar CH_4, 654 μmol H_2O_2, 20 mL H_2O, 50 ℃, 6 h	0.01	1.2	57.0	6
PdO/Pd–WO_3	100 mg samples, CH_4 and H_2O flow, 3h	-	-	91.6	7
Ce-UiO-Cu(OH)	3.7 mg samples, 6 bar O_2, 30 bar CH_4, 8 mL H_2O, 115 ℃, 40 h	0.93	155.0	99.9	8
(Pt/NPW)/TiO_2	50 mg samples, 1 bar CO, 10 bar CH_4, 10 mL H_2O, 25 ℃, 400W Hg-Xe lamp, 60 h	0.03	5.7	90.6	9
Ir complex /SBA-15	15 mg samples, 4 bar O_2, 5 bar CO, 19 bar CH_4, 15 mL H_2O, 150 ℃, 3 h	0.06	10.2	65.6	10
Rh_1/pMOF	20 mg samples, 4 bar O_2, 5 bar CO, 15 bar CH_4, 20 mL H_2O; 150 ℃, 3 h	0.43	70.9	83.8	11
Pd_1-ZSM-5	25 mg samples, 25 °C, 15 mL 0.6 M H_2O_2, 30 bar CH_4, 20 bar CO, 0.5 h	0.01	1.3	78.3	12
Ni-ZSM-5	20 mg samples, 5 bar of CO, 25 bar CH_4, 654 μmol H_2O_2, 20 mL H_2O, 50 ℃, 10 h	0.02	2.9	82.3	13
Pt-HPW-TiO_2	1 g samples, 1 bar CO, 15 bar CH_4, 0.1 mL H_2O; 175 ℃, 2 h	1.06	177.3	99	**This work**

Table S2. *ICP-OES analysis of metal content*

Sample	Co (wt. %)	Cu(wt. %)	Pd(wt. %)	Rh(wt. %)	Pt(wt. %)	W(wt. %)
**Pt-HPW-TiO_2**	-	-	-	-	0.202	0.159
**Pt-HPW-TiO_2 after reaction**	-	-	-	-	0.174	0.150
**Pt-HPW-TiO_2 after regeneration**	-	-	-	-	0.167	0.147
**Rh-HPW-TiO_2**	-	-	-	0.247	-	
**Pd-HPW-TiO_2**	-	-	0.226	-	-	
**Cu-HPW-TiO_2**	-	0.162	-	-	-	
**Co-HPW-TiO_2**	0.194	-	-	-	-	

1. Periana, R. A.; Mironov, O.; Taube, D.; Bhalla, G.; Jones, C., Catalytic, Oxidative Condensation of CH₄ to CH₃COOH in One Step via CH Activation. *Science* **2003,** *301* (5634), 814-818.
2. Narsimhan, K.; Michaelis, V. K.; Mathies, G.; Gunther, W. R.; Griffin, R. G.; Román-Leshkov, Y., Methane to Acetic Acid over Cu-Exchanged Zeolites: Mechanistic Insights from a Site-Specific Carbonylation Reaction. *Journal of the American Chemical Society* **2015,** *137* (5), 1825-1832.
3. Shan, J.; Li, M.; Allard, L. F.; Lee, S.; Flytzani-Stephanopoulos, M., Mild oxidation of methane to methanol or acetic acid on supported isolated rhodium catalysts. *Nature* **2017,** *551* (7682), 605-608.
4. Tang, Y.; Li, Y.; Fung, V.; Jiang, D. E.; Huang, W.; Zhang, S.; Iwasawa, Y.; Sakata, T.; Nguyen, L.; Zhang, X.; Frenkel, A. I.; Tao, F. F., Single rhodium atoms anchored in micropores for efficient transformation of methane under mild conditions. *Nat Commun* **2018,** *9* (1), 1231.
5. Qi, G.; Davies, T. E.; Nasrallah, A.; Sainna, M. A.; Howe, A. G. R.; Lewis, R. J.; Quesne, M.; Catlow, C. R. A.; Willock, D. J.; He, Q.; Bethell, D.; Howard, M. J.; Murrer, B. A.; Harrison, B.; Kiely, C. J.; Zhao, X.; Deng, F.; Xu, J.; Hutchings, G. J., Au-ZSM-5 catalyses the selective oxidation of CH4 to CH3OH and CH3COOH using O2. *Nature Catalysis* **2022,** *5* (1), 45-54.
6. Wu, B.; Lin, T.; Lu, Z.; Yu, X.; Huang, M.; Yang, R.; Wang, C.; Tian, C.; Li, J.; Sun, Y.; Zhong, L., Fe binuclear sites convert methane to acetic acid with ultrahigh selectivity. *Chem* **2022,** *8* (6), 1658-1672.
7. Zhang, W.; Xi, D.; Chen, Y.; Chen, A.; Jiang, Y.; Liu, H.; Zhou, Z.; Zhang, H.; Liu, Z.; Long, R.; Xiong, Y., Light-driven flow synthesis of acetic acid from methane with chemical looping. *Nat Commun* **2023,** *14* (1), 3047.
8. Antil, N.; Chauhan, M.; Akhtar, N.; Kalita, R.; Manna, K., Selective Methane Oxidation to Acetic Acid Using Molecular Oxygen over a Mono-Copper Hydroxyl Catalyst. *Journal of the American Chemical Society* **2023,** *145* (11), 6156-6165.
9. Dong, C.; Marinova, M.; Tayeb, K. B.; Safonova, O. V.; Zhou, Y.; Hu, D.; Chernyak, S.; Corda, M.; Zaffran, J.; Khodakov, A. Y.; Ordomsky, V. V., Direct Photocatalytic Synthesis of Acetic Acid from Methane and CO at Ambient Temperature Using Water as Oxidant. *Journal of the American Chemical Society* **2023,** *145* (2), 1185-1193.
10. Li, H.; Fei, M.; Troiano, J. L.; Ma, L.; Yan, X.; Tieu, P.; Yuan, Y.; Zhang, Y.; Liu, T.; Pan, X.; Brudvig, G. W.; Wang, D., Selective Methane Oxidation by Heterogenized Iridium Catalysts. *J Am Chem Soc* **2023,** *145* (2), 769-773.
11. Li, H.; Xiong, C.; Fei, M.; Ma, L.; Zhang, H.; Yan, X.; Tieu, P.; Yuan, Y.; Zhang, Y.; Nyakuchena, J.; Huang, J.; Pan, X.; Waegele, M. M.; Jiang, D.-e.; Wang, D., Selective Formation of Acetic Acid and Methanol by Direct Methane Oxidation Using Rhodium Single-Atom Catalysts. *Journal of the American Chemical Society* **2023,** *145* (20), 11415-11419.
12. Xu, W.; Liu, H.-X.; Hu, Y.; Wang, Z.; Huang, Z.-Q.; Huang, C.; Lin, J.; Chang, C.-R.; Wang, A.; Wang, X.; Zhang, T., Metal-Oxo Electronic Tuning via In Situ CO Decoration for Promoting Methane Conversion to Oxygenates over Single-Atom Catalysts. *Angewandte Chemie International Edition* **2024,** *63* (16), e202315343.
13. Liu, J.; Wei, Y.; Li, R.; Liu, Y.; Yu, H.; Zhou, X.; Wu, B.; Lin, T.; Zhong, L., Isolated Ni sites anchored on zeolites for direct synthesis of acetic acid from methane oxidative carbonylation. *Applied Catalysis B: Environment and Energy* **2024,** *350*, 123951.
